# Migration health research in Norway: a scoping review

**DOI:** 10.1177/14034948211032494

**Published:** 2021-10-05

**Authors:** Johanna Laue, Esperanza Diaz, Linda Eriksen, Torsten Risør

**Affiliations:** 1 Department of Community Medicine, UiT The Arctic University of Norway, Norway; 2 Department of Global Public Health and Primary Care, University of Bergen, Norway; 3 Unit for Migration and Health, Norwegian Institute of Public Health, Norway

**Keywords:** Emigrants and Immigrants, Refugees, Health, Review, Population, Methods, Norway, Research

## Abstract

*Aims:* To provide an overview of published research on migration and health conducted in Norway and identify gaps in the research field. *Methods:* Applying a scoping review methodology, we searched Medline for articles on migration health in Norway published between 2008 and 2020, and assessed them according to research topic, methodology, user-involvement and characteristics of the populations studied (country or area of origin, type of migrant/immigrant status). *Results:* Of the 707 articles retrieved, 303 met the inclusion criteria. Most studies (77%) were within the clinical disciplines reproductive health, mental health, infectious diseases and cardiovascular diseases, or on socio-cultural aspects and the use of healthcare services. One third of the papers (36%) pulled participants from various geographic backgrounds together or did not specify the geographic background. Among those who did so, participants were mostly from The Middle East, South and Southeast Asia and sub-Saharan Africa. Only 14% of the articles specified the type of migrant/immigrant status and those included refugees, asylum seekers and undocumented migrants. A total of 80% of the papers used quantitative methods, of which 15 described an intervention; 15 papers (5%) described different types of user-involvement. ***Conclusions:* Our findings suggest gaps in research related to migrant subgroups, such as those from Eastern-Europe and labour and family reunification migrants. Future studies should further investigate the self-identified health needs of different migrant groups, and might also benefit from a methodological shift towards more intervention studies and participatory approaches.**

## Introduction

### Migration trends

In the last 15 years, international migration has increased to include today approximately 272 million people, or 3.5% of the world’s population [1]. These migrants are distributed unevenly between and within countries. In Norway, the number of migrants and their descendants has increased tenfold since the 1950s [2]. In 2020, migrants and their descendants accounted for approximately 15% of the Norwegian population, with backgrounds from a variety of different countries or regions [2]. Polish labour migrants account for the largest migrant group in Norway [2]. These diverse populations also have heterogenous reasons for migration. Both globally and in Norway, labour migrants account for the largest percentage of international migrants, followed by people who immigrate due to family reunification, refuge and education [1,2].

### The relationship between migration and health is complex and dynamic

The health of migrants is important to consider in a society, both from a human rights perspective and because it affects the host society as a whole [3]. In Norway, as internationally, the health status of migrants differs from the health status of the host population [4,5]. These differences are complex and simple cause–effect explanations are insufficient. Theories and hypotheses to explain differences in health status and health care use between migrant and host populations include ‘Healthy migrant effect’, ‘Allostatic load’, ‘Acculturation’ and ‘Genetics’ [6]. Clearly, the health of migrants is influenced by various aspects such as reasons for, and nature of, migration, origin countries and conditions of the places they came from and travelled through, as well as individuals’ initial health condition [7]. Importantly, migrants’ health is further influenced by conditions in their new host country: social inequalities, language difficulties, unstable living conditions, discrimination, ‘cultural crash’ or the ability of health care systems to meet the migrants’ needs [3].

### Research on migration and health

Research on migrant health is important to inform policy makers and healthcare personnel about how to improve the health of migrants, and may also inform public discourse [5,8]. The increased international attention paid to this field in the last two decades is reflected in a growing number of publications [8], including evidence produced by the relatively newly launched *Lancet Commission on Migration and Health* [5].

Collecting and mapping available international research on migration and health can help reveal gaps in our understanding, and suggest future research activities to address health needs of international migrants to a larger extent [5,8]. However, the health of migrants varies across national contexts. This depends on many factors associated with major migrant groups, host societies and their interaction [9]. National research activity should therefore include health needs of migrants in a specific national context. There are already a few reviews on migration health-related research in Norway [3,7,10–12]. These focus mostly on different health challenges migrants in Norway have, or include only certain migrant groups. None of them provide an overview over characteristics of the actual research activity and resulting research gaps in Norway. Such an overview can help to facilitate more effectively coordinated future research in the field in Norway, and can be an inspiration for other countries to map research activity on migration health in their own context.

## Aim

The overall aim of this study is to provide an overview of peer-reviewed research on migration and health conducted in Norway in the last 12 years. Specifically, we aim to describe the research field thematically in terms of research topics, populations studied, user-involvement and methodologies used.

## Methods

This is a scoping review, which is useful to rapidly map existing evidence concerning a research area, to examine the nature, range and extent of the research activity and to identify gaps in knowledge [13]. We applied the following steps, as described by Arksey and O’Malley [13]: Identifying the research question, identifying relevant studies, study selection, charting the data, summarizing the results.

Our research question was ‘Which and what type of evidence is available in Norway regarding migrant health?’. As a next step, we designed a search strategy built on Medical Subject Headings (MeSH) and relevant free text words, and searched in Medline including abstracts, key words and titles. Basically, we combined MeSH terms and free text words to cover different types of migrant groups (‘Emigrants’, ‘Immigrants’, ‘Migrants’, ‘Ethnicity’, ‘Ethnic group’, ‘Multiethnic’, ‘Minority group’, ‘Ethnology’, ‘Refugees’, ‘Transients and migrants’, ‘Asylum seekers’, ‘Labour Migrants’, ‘Undocumented Migrants’, ‘Unaccompanied minors’) with the free text word ‘Norway’. See Figure S1 in the supplemental material for the complete search strategy. We conducted the systematic search on 18 October 2018 in Medline to identify all publications that concern health related topics for all types of international migrants in Norway as defined by the authors of the retrieved publications. Limiting the search to Medline only was chosen due to capacity reasons, and we are aware that a search in other databases, such as Web of Science, could have provided additional articles, especially from social sciences, using qualitative methods. The search was limited to the period 2008 to the date of search, as we expected the number of publications in the field to be largest within the last decade [8]. We repeated the search on 22 September 2020 using the same search strategy but limited to publications between 2018 and date of search.

Further, we read the title and abstract of all retrieved documents to assess eligibility, applying the following inclusion criteria:

- research with a focus on health-related topics relevant to migrants’ health status or healthcare for migrants;
- research conducted in Norway;
- first author belongs to a Norwegian university, school or research institution; and
- publication in English or Norwegian language.

We read through titles, keywords, abstracts and method sections of included articles and sorted them by research topic, population studied (country or area of origin, gender, age, type of migrant) and methodology used (quantitative, qualitative, mixed methods, intervention). We also assessed whether or not involvement of users in the research was described in the papers. We defined ‘users’ as all other types of persons/institutions/organisations except researchers, and ‘involvement’ as all types of activities that bring the users’ perspective into the research. We used a ‘bottom-up’ approach for assessing research topics and populations studied, meaning that we developed analytical labels as they emerged from the studies. For studies fitting more than one category (for instance mental health AND use of healthcare services, or cardiovascular risk factor AND endocrine diseases), we chose the topic that was the main priority in the study as described in its aim and conclusion. When still in doubt, the thematic focus of the journal it was published in determined the category. For instance, if a study on mental health and use of healthcare services was published in a journal dealing with psychiatry, mental health was chosen as category. In terms of study participants’ geographical background, we grouped studies that did not specify the geographical background of participants with those that did so but did not report their findings/conclusion according to geographical background into the category ‘various’. We categorised studies including participants from one or several countries or geographic regions, and studies reporting findings/conclusions to the participants’ geographical background, according to how this was described in the papers. For assessing the geographical background quantitively, we based calculations on country representations instead of number of studies, as many studies included participants from more than one country/geographical region. We pre-defined categories regarding research methodology as either quantitative, qualitative, mixed methods or review, whether or not the study was an intervention study and whether or not the involvement of users was described in the methods’ section.

## Results

**Figure 1.**
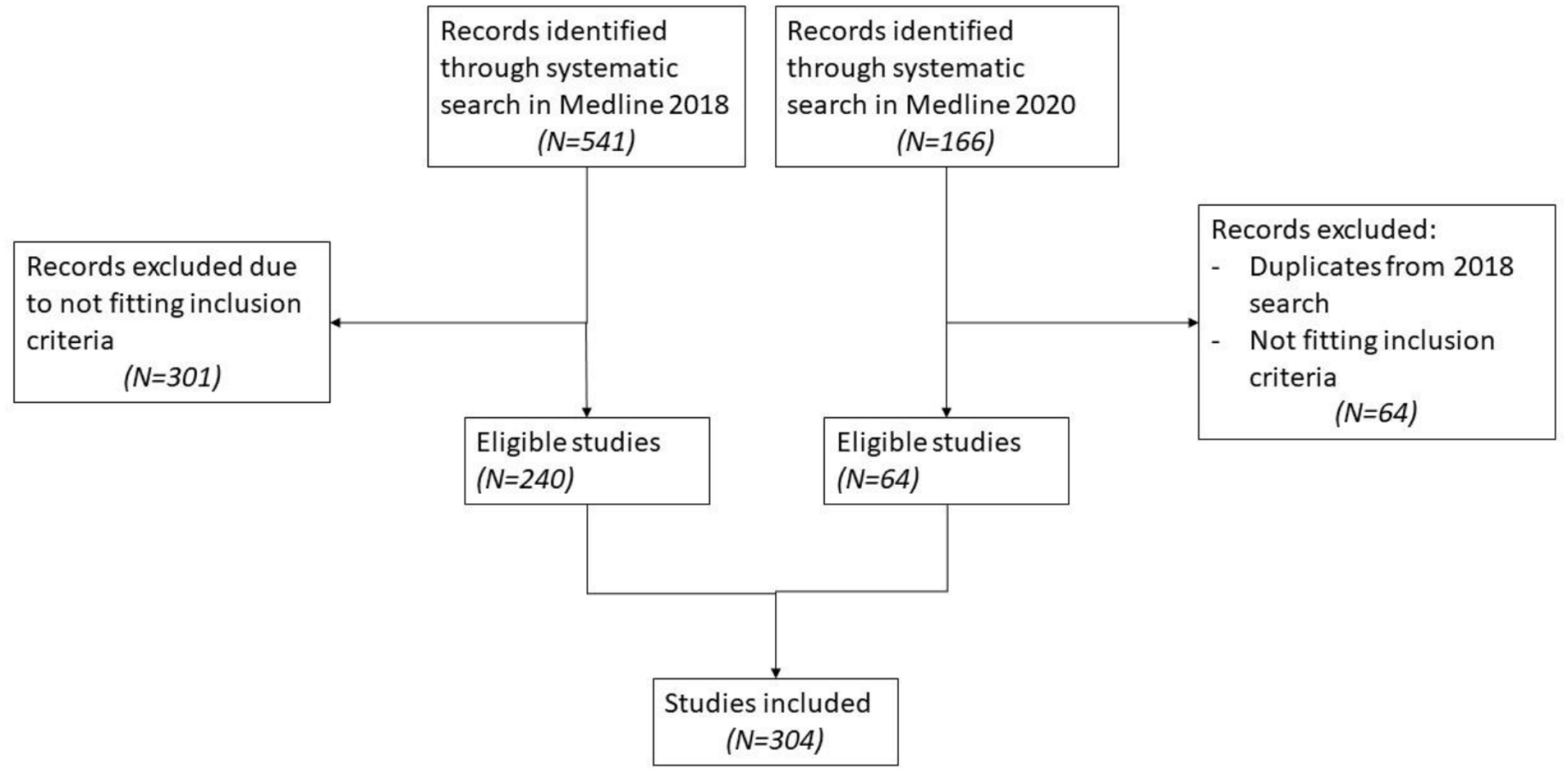
Inclusion process.

Figure 1 shows the process of inclusion and exclusion of papers. We retrieved 541 articles from the search in Medline in 2018, of which 239 documents were included in the study. The search in Medline in 2020 revealed an additional 166 articles, of which 64 were included in the study. In total, 303 peer-reviewed articles were included (see Table I in supplementary material).

### Research topics

**Figure 2.**
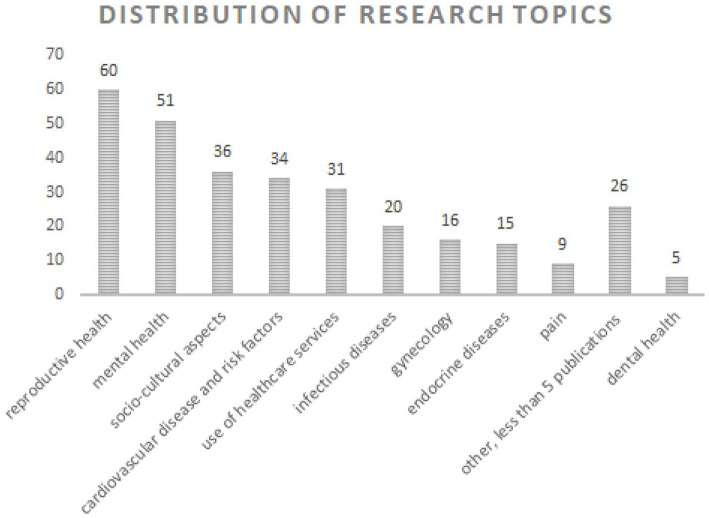
Research topics.

The bottom-up assessment of research topics resulted in 22 categories (see Figure 2). Most studies covered the clinical disciplines reproductive health (20%, *n* = 60), mental health (17%, *n* = 51), cardiovascular diseases/risk factors (11%, *n* = 34), infectious diseases (7%, *n* = 20), gynaecologic health problems (5%, *n* = 16) and endocrine diseases (5%, *n* = 15). The two other dominating categories included a variety of topics related to understanding patients’ or providers’ perspectives and social and cultural aspects relevant for patient care (‘Socio-cultural aspects’ 12%, *n* = 36) and to use of healthcare services among migrant patients (‘Use of healthcare services’ 10%, *n* = 31). The thematic areas with fewer than 10 publications were ‘Pain’ (3%, *n* = 9), ‘Dental health’ (2%, *n* = 5), ‘Cancer’ (1%, *n* = 4), ‘Neurologic disorders’ (1%, *n* = 4), ‘Substance abuse’ (1%, *n* = 4), ‘Nutrition’ (1%, *n* = 3), ‘Multimorbidity’ (0.7%, *n* = 2), ‘Quality of life’ (0.7%, *n* = 2), ‘Otolaryngology’(0.3%, *n* = 1), ‘Forensics’ (0.3%, n=1), ‘Geriatrics’ (0.3%, *n* = 1), ‘Orthopedics’ (0.3%, *n* = 1), ‘Vaccinations’ (0.3%, *n* = 1) and ‘Use of medications’ ‘(0.3%, *n* = 1).

#### Reproductive health

Predominant topics within this field were pregnancy-related conditions or complications, such as gestational diabetes, lifestyle factors/overweight and physical activity, pre-eclampsia, hypertension, hyperemesis, depression, *Helicobacter pylori* infection related to pregnancy, incontinence and deficiencies of vitamin D, iron and folic acid. Other topics covered delivery complications (stillbirth, preterm labour, termination and perinatal mortality), contraception and breastfeeding. See Table I.

#### Mental health

Studies on mental health dealt mostly with either the prevalence of various mental health conditions or the role of various risk factors for developing mental illness (discrimination, childhood trauma, asylum process, stressful life experiences, acculturation hassles). There were also studies on coping strategies/resilience/social support, one study on the validity of screening for psychiatric disorders and a review on what is known and not known about mental health problems among migrants. See Table I.

#### Cardiovascular diseases and risk factors

Studies in this category dealt mostly with prevalences or moderating risk factors for cardiovascular diseases (diabetes type 2, physical activity, overweight). Other studies explored barriers to healthy eating and there were two randomised controlled trials (RCTs) on nutritional habits and physical activity (see Table I).

#### Infectious diseases

This category included first and foremost studies on tuberculosis among migrants (related mostly to screening). There were a few studies on aspects related to sexually transmitted diseases, and one study on methicillin-resistant *Staphylococcus aureus* MRSA (see Table I).

#### Endocrine disorders

Studies in this category dealt mostly with vitamin D related topics (vitamin D status, nutritional rickets and vitamin D supplementation) including two RCTs on the effect of vitamin D on muscle strength and thyroid autoimmunity, respectively. Other studies dealt with type I diabetes (see Table I).

#### Gynaecology

Most studies examined different aspects of female circumcision. Some focused on migrant women’s participation at cervical cancer or breast cancer screening programs, including one RCT to increase attendance rates for cervical screening programs (see Table I).

#### Pain

This topic included studies on use of analgesic drugs, chronic pain and long-term sick leaves as well as effects of vitamin D on musculoskeletal pain. There was also a RCT protocol on treating pain disorders among migrants (see Table I).

#### Dental health

Studies in this category looked mostly at children’s dental health, especially in relation to their parents’ attitudes (see Table I).

#### Socio-cultural aspects

A variety of topics were found in this category, including cultural barriers to providing good health care from the perspectives of healthcare personnel and to the influence of culture on the use of medication, managing illness, health literacy, navigating the healthcare system and on tailoring health information to migrants. Moreover, it included topics related to the Norwegian welfare state and experiences of living in the Norwegian society (see Table I).

#### Use of healthcare services

These studies assessed either migrants’ utilisation of different healthcare services compared with the native population, with different aspects regarding access to healthcare services (barriers/facilitators, motivations) or experiences of migrants with healthcare (see Table I).

### Populations studied

#### Country or region of origin

As many as 110 studies (36%) did not specify the country background of their study participants or combined various countries/regions without relating their results to the participants’ geographical background (‘various’ geographical background). Approximately one-quarter of all studies (23%, *n* = 69) had participants from one country only, while 37 studies (12%) included people from more than one country in various combinations. Another 23% (*n* = 71) described the background of their participants in larger geographical regions or continents without further specification of the country. Moreover, 4 studies described the background of their participants as high-income, middle-income or low-income countries, and 13 studies were on perspectives of Norwegian healthcare professionals on migrant health related topics (including 1 which studied both the perspectives of migrants and Norwegian healthcare personnel).

**Figure 3.**
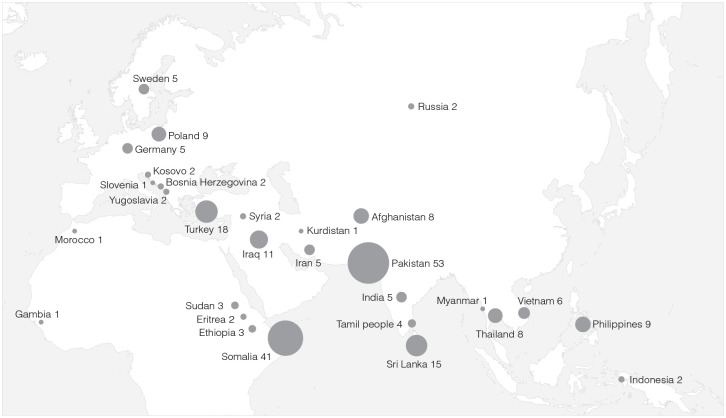
Country background.

In the 106 studies specifying participants’ country of origin, 29 countries were represented at least once (either alone or in combination with other countries). Migrants from Pakistan (*n* = 53) and Somalia (*n* = 41) were most often represented, followed by Turkey (*n* = 18), Sri Lanka (*n* = 15), Iraq (*n* = 11), the Philippines (*n* = 9), Poland (*n* = 9), Thailand (*n* = 8), Afghanistan (*n* = 8), Vietnam (*n* = 6), India (*n* = 5), Iran (*n* = 5), Sweden (*n* = 5), Tamil people (*n* = 4), Sudan (*n* = 3), Ethiopia (*n* = 3), Indonesia (*n* = 2), Syria (*n* = 2), Kosovo (*n* = 2), Eritrea (*n* = 2), Germany (*n* = 2), Russia (*n* = 2), Yugoslavia (*n* = 2), Chile (*n* = 2), Bosnia and Herzegovina (*n* = 2), Myanmar (*n* = 1), Morocco (*n* = 1), Gambia (*n* = 1), Slovenia (*n* = 1) and Kurdistan (*n* = 1) (see Figure 3).

When dividing origin countries into larger geographic regions (as defined by the United Nations Statistics Division [14], we find that Asian migrants account for 66% of the total 224 representations of geographic regions or continents (Southern Asia 40%, *n* = 90;Western Asia 14%, *n* = 32;South Eastern Asia 12%, *n* = 26), followed by African migrants (Sub-Saharan Africa 22%, *n* = 50;Northern Africa 0.4%, *n* = 1), Europe (Eastern Europe 5%, *n* = 11;Northern Europe 2%, *n* = 5;Southern Europe 2%, *n* = 5;Western Europe 1%, *n* = 2) and South America (1%, *n* = 2).

**Figure 4.**
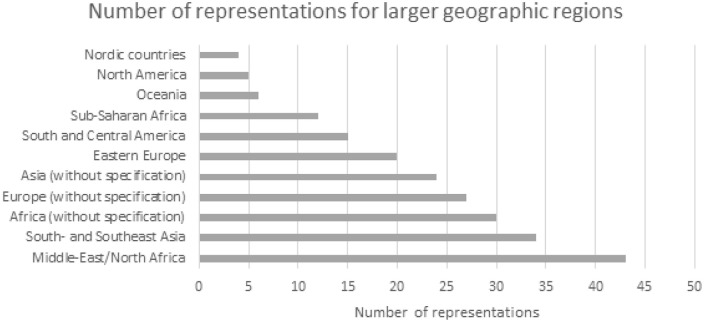
Number of representations for larger geographic regions based on descriptions in the papers.

There were 71 studies describing the geographical background of study participants in one or several larger regions or continents. Altogether, we found 11 different geographic regions described alone or in various combinations. We sorted them based on how the regions were described in the publications. Asia was the region most often represented (see Figure 4).

Altogether, we found 20 representations of the terms ‘western’ or ‘non-western’ for describing the geographic origin of study participants (Western countries *n* = 8, non-Western countries *n* = 12). Four studies (in total 12 representations) described their participants’ background in terms of income and one study described its participants as being from ‘developing countries’.

#### Gender and age

Most studies (61%, *n* = 183) included adults of both genders. Approximately one-quarter (26%, *n* = 79) included women only, with many studies on Pakistani and Somali women. We found 36 studies (12%) on children or adolescents of both genders, and 4 studies (1%) that included (Pakistani) men only.

#### Type of migrants/immigrant status

We classified for this paper type of migrant/immigrant status as both the reason for migration and the legal status of the migrants. Only 42 studies (14%) provided information regarding the immigrant status of their participants. All but two of them included people with one immigrant status only (refugees *n* = 17, asylum seekers *n* = 10, unaccompanied minors *n* = 10, undocumented migrants *n* = 3), and two studies included both refugees and asylum seekers.

### Methodology

Of the 303 studies, 242 (80%) used quantitative methods, 55 (18%) qualitative methods, 4 (1%) mixed methods and 2 studies (0.1%) were reviews. There were 12 papers describing results of two RCTs [15–26], and 1 of a non-randomized controlled trial [27]. Two papers were protocols of RCTs [28,29], and one paper described a new municipal care program [30]. We found a description of user-involvement in 15 papers (5%), based on 13 individual studies. The type of user-involvement varied from performing qualitative studies with users or collaboration with users to inform the researchers about challenges as a basis for an intervention [15,25–27,30,31], collaboration to develop an intervention [28], or to learn more about the subject of investigation, i.e. female genital mutilation [32]. In five papers, users were involved as research assistants to recruit participants or collect data [33–37]. In one study, users were involved in the whole research process (‘including the adaption and translation of tools, recruitment and data collection, transcription and translation of data, and primary data analysis’ [38].

## Discussion

### Summary of results

This scoping study provides an overview of research conducted in the field of ‘migration health’ in Norway. We found that studies focus thematically on a few clinical disciplines (reproductive health, mental health, infectious diseases and cardiovascular diseases and risk factors), but also on socio-cultural aspects and the use of healthcare services. Most studies include migrants from South and Southeast Asia and sub-Saharan Africa, often pooling participants from several countries together. Many studies do not specify either the participants’ geographical background or immigrant status. Most studies are descriptive, very few are intervention studies and few papers describe user involvement in their methods section.

### Are we addressing the real health needs of migrants?

Our finding that migrant health research is dominated by certain clinical fields is in line with two previous reviews from Norway [3,10], one review on existing research on migration health from the Republic of Ireland [39] and one bibliometric analysis on migration health research on a global basis [8]. The clinical fields of mental health or infectious diseases may indeed cover important health needs of some migrants, as for instance tuberculosis is more prevalent among some groups of migrants from low-income countries, and mental health issues are truly a great challenge for migrants with a flight background or victims of human trafficking [5]. Yet, existing studies suggest that it is rather musculoskeletal complaints, pain, stress, psychosocial conditions and complex unexplained health problems that are among the most prevalent health problems of many migrants. [40–43]. While acknowledging that these less studied health problems may not affect all migrants, they have, however, great societal relevance, as they for instance can negatively influence migrants’ participation in the labour market [41,44]. We found very few studies which explicitly address these complex health problems, even though studies within some of our categories (‘mental health’ or ‘socio-cultural aspects’) also address stress and psychosocial aspects. It is difficult to make a clear statement about what the most significant health needs of migrants actually are, given the small number of studies [40–43] that explore self-rated health, due to the migrant population’s heterogeneity, and since what is perceived as ‘most significant’ can vary between whether one applies a public health perspective or the perspective of the individual. However, our findings indicate a mismatch between existing research priorities and significant health needs of migrants and societal challenges related to migrant health in Norway. One explanation may relate to lack of information on migrant related aspects in existing health registries. For instance, The Tromsø Study [45] with its assessment of health related factors and conditions for a whole municipality over five decades does not include information about nationality, language or ethnicity (besides Sami and Finnish Origin). It is also a general challenge in health research and clinical care that some health problems (such as diffuse and complex health problems which do not easily fit into disease categories) receive less attention than others without this being justifiable by the individual or societal illness burden [46]. We suggest that to develop systematic and ethically sound ways to ensure migrant health research in Norway and make it more ‘needs-driven’ [47] is an important task for the research community.

A considerable number of studies address socio-cultural aspects or use of healthcare services. These are important topics to address to fully understand health challenges of migrants. Importantly, compared with the scoping review from The Republic of Ireland which finds that most research relates to social determinants of health, public health preparedness and health system adaptations [39], Norwegian studies on socio-cultural aspects focus mostly on ‘culture’ as a determinant of health and healthcare. However, there is a need for better consideration of structural and socio-economic factors such as education or income as interdependent but different variables linked to health and disease in order to deepen our understanding of the determinants of health for migrants as much more than being a question of ‘culture’. Having a focus on ‘culture’, particularly the migrants’ culture, as the main barrier to health equity entails the risk of narrowly focusing on migrants’ culturally influenced behavior to improve health, and of placing the responsibility for good health with the individual only while overlooking structural factors [48]. In fact, the interventions we found in our review aim mostly at changing migrants’ behavior [26,28,30], increase their health literacy [23] or test treatments [19,29], while only one intervention turned around to address health service organization [15]. Also, as pointed out earlier [3,48], increasing attention to societal factors such as discrimination in future research could expand the existing knowledge base and serve as an important step towards reducing health disparities and achieving equitable services for migrant populations.

### Methodological aspects

The dominance of descriptive studies, mostly quantitative, is in line with the reviews from Norway [3,10] and Ireland [39]. The use of methodological approaches consistent with a positivist epistemology comes with the risk of toning down complexities within the field, here migration health, and can leave out important relational aspects, such as how people’s social reality is shaped, and how they understand their own and others’ actions [49]. Lack of research that provides necessary complementary perspectives and a deeper understanding of migrants’ health outcomes in Norway has earlier been identified and criticized [3].

Few papers describe involvement of users, and ways in which users were involved were mostly quite peripheral (only in the first stages of the research process or to collect data). Thus, except for one study, user-involvement in Norwegian migration health research seems to rather reflect a ‘tokenistic’ [50] way to involve users instead of a true commitment to involve users in the whole research [50]. Underrepresentation of migrants and ethnic minorities in Norwegian research activity has been described recently, and the authors identify several barriers to user-involvement that includes all stages of the research process [51]. According to our findings, knowledge on users’ perspectives (both migrants and healthcare providers) seems to be generated almost solely through qualitative studies, which can indeed give insight into migrants’ and healthcare providers’ needs, yet may not be able to benefit from the advantages of ‘true’ user-involvement [50].

We also found very few intervention studies. Methods that go beyond observation and description are needed to be able to adapt services to multicultural populations and to secure that the measures are in line with the needs [52]. Overall, our findings suggest that research communities should shift their methodological focus towards more intervention studies and participatory approaches through which migrants themselves can contribute in various stages of research processes [52,53].

### Disproportionate focus on a few groups of migrants

We found an overly focus on migrants from South and Southeast Asia, sub-Saharan Africa and the Middle East. Yet, migrants from Eastern Europe and other European countries are the largest migrant groups in Norway, often immigrating as labour migrants or due to family reunification [2]. We found few studies on, for instance, Polish labour migrants, although many of them indeed have health problems and can experience significant barriers to accessing health care [4,42,54–58]. Health of labour migrants, especially those from less affluent countries, who often work in sectors with high exposure to occupational hazards and have jobs characterized by poor working conditions and limited legal rights [55,57–59], should be a priority in future research on migrant health. There is also little research that specifically addresses other large and small migrant groups, such as family reunification migrants or undocumented migrants.

Many studies included migrants with various geographic backgrounds or immigrant status or pooled data from participants from different backgrounds together. This finding is in line with a bibliometric analysis of global migration health research [8]. On the one hand, a generalising approach to understanding health issues can add important information about overall aspects, and pooling data may help generate statistical power. It can in fact be adequate to pool migrant groups when the study’s aim is to, for instance, address factors that are known to significantly affect several groups of immigrants in Norway. On the other hand, when the majority of studies is based on data with little granularity (for instance, when grouping migrants from one continent together); this will result in a knowledge base which can hide important details and differences between subgroups of migrants [60]. Lack of migration specific details in for example registry data has been pointed our previously [60].

### Strengths and limitations

We used a broad search strategy and followed the methodological steps rigorously. Yet, we limited our search to one database only and we may therefore have missed some publications, especially regarding psychosocial aspects of health or studies from the social sciences using qualitative methods to a larger extent, which could be indexed in other, non-medical, databases. As a result, the research gaps may be narrower than we found in this review. Moreover, our search does not include reports from national bodies such as The Norwegian Institute of Public Health, which are not published in research databases. However, they usually build upon research papers, and we do not think that additional searches would have altered the overall picture of the research landscape significantly. We consider our bottom-up strategy for categorising the studies as most reasonable for developing an understanding of what is actually researched. This approach differs also from previous reviews, which mostly used pre-defined categories for assessing research [3,39], and thus, adds to existing knowledge on the research field. Yet, other researchers might have developed other categories, especially for the category of ‘system/culture/society/language’, and might have interpreted the description of study participants and user-involvement differently, both of which could have led to slightly different results.

## Conclusion and implications

Our findings suggest significant gaps in research related to migrant subgroups, such as those from Eastern Europe and labour and family reunification migrants. Future studies should explore and further investigate the self-identified health needs of different migrant groups. A methodological shift in migration health research towards more intervention studies, and participatory approaches could be useful to better understand the complexities in migration health issues and to effectively reduce the health disparities that still exist between migrants and the Norwegian population.

## Supplemental Material

sj-pdf-1-sjp-10.1177_14034948211032494 – Supplemental material for Migration health research in Norway: a scoping review Click here for additional data file. Supplemental material, sj-pdf-1-sjp-10.1177_14034948211032494 for Migration health research in Norway: a scoping review by Johanna Laue, Esperanza Diaz, Linda Eriksen and Torsten RisØr in Scandinavian Journal of Public Health
