# Supplementary material for: Migration health research in Norway: a scoping review
Source: Scand J Public Health. 2021 Oct 5;51(3):381–90. doi: 10.1177/14034948211032494 (PMC10251465; doi:10.1177/14034948211032494)
Supplement: sj-pdf-1-sjp-10.1177_14034948211032494 – Supplemental material for Migration health research in Norway: a scoping review [file sj-pdf-1-sjp-10.1177_14034948211032494.pdf]

Table I. Characteristics included papers.

Reference	Characteristics of study participants			Methodology	Intervention (yes/no – not relevant)	User-involvement described (yes/no – not relevant)
	Geographic background	Gender/age group (f=female; m=male; c=child; a=adolescent)	Immigrant status (n.n.=not described –=not relevant)			
Reproductive health						
Bjerke et al. 2008[1]	Pakistan	f	n.n.	quantitative	no	no
Vikanes et al. 2008[2]	Turkey, Middle East, North Africa, Africa, Iran, Pakistan, India, Sri Lanka, Central and South America	f	n.n.	quantitative	no	no
Vangen et al. 2008[3]	various	f/m	n.n.	quantitative	no	no
Grjibovski et al. 2009[4]	Pakistan	f/m	n.n.	quantitative	no	no
Jenum et al. 2010[5]	Europe, Asia, Middle East, Africa, South America	f	n.n.	quantitative	no	no
Singh et al. 2010[6]	Pakistan, India	f/m	n.n.	quantitative	no	no
Bjerke et al. 2011[7]	Pakistan	f/m	n.n.	quantitative	no	no
Bø et al. 2012[8]	Europe/North America, Asia, Middle East, Africa	f	n.n.	quantitative	no	no
Lyberg et al. 2012[9]	Norwegian healthcare providers	f/m	–	qualitative	no	no
Mørkrid et al. 2012[10]	Western Europe, South Asia, Middle East, East Asia	f	n.n.	quantitative	no	no
Sachse et al. 2012[11]	various	f/m	n.n.	quantitative	no	no
Tønnessen et al. 2013[12]	India	f/m	n.n.	quantitative	no	no
Brekke et al. 2013[13]	various	f	n.n.	quantitative	no	no
Garnweidner et al. 2013[14]	Africa, Asia	f	n.n.	qualitative	no	no
Naimy et al. 2013[15]	Pakistan, Vietnam, Somalia, Sri Lanka, Philippines, Iraq, Thailand, Afghanistan	f/m	n.n.	quantitative	no	no
Vikanes et al. 2013[16]	various	f	n.n.	quantitative	no	no
Sletner et al. 2013[17]	Western Europe, Asia, Middle East, Africa, South/Central Asia	f/m	n.n.	quantitative	no	no
Sletner et al. 2014[18]	various	f	n.n.	quantitative	no	no
Berntsen et al. 2014[19]	South Asia, Middle East	f/m	n.n.	quantitative	no	no
Sørbye et al. 2014[20]	Pakistan	f	n.n.	quantitative	no	no
Sørbye et al. 2014[21]	Iraq, Pakistan, Somalia, Philippines, Sri Lanka, Vietnam	f	n.n	quantitative	no	no
Omland et al. 2014[22]	various	f	n.n.	quantitative	no	no
Shakeel et al. 2015[23]	Western Europe, Middle East, South Asia	f/m	n.n.	quantitative	no	no
Sørbye et al. 2015[24]	Pakistan, Vietnam, Philippines, Poland, Sri Lanka, Yugoslavia, Slovenia, Iraq, Somalia, Thailand, Turkey	f	n.n.	quantitative	no	no
Naimy et al. 2015[25]	Pakistan, Vietnam, Somalia, Sri Lanka, Philippines, Iraq, Thailand, Afghanistan	f	n.n.	quantitative	no	no
Viken et al. 2015[26]	South America, Europe, Middle East, Asia, Africa	f	n.n.	qualitative	no	no
Bakken et al. 2015[27]	Asia, Africa	f	n.n.	quantitative	no	no
Bakken et al. 2015[28]	Somalia, Iraq, Afghanistan, Kosovo	f	n.n.	quantitative	no	no
Sletner et al. 2015[29]	Europe, South Asia, East Asia, Middle East/North Africa	f/m	n.n.	quantitative	no	no
Richardsen et al. 2016[30]	Western Europe, South Asia, Middle East	f	n.n.	quantitative	no	no
Sommer et al. 2016[31]	various	f	n.n	quantitative	no	no
Waage et al. 2016[32]	Western Europe, Eastern Europe, South Asia, Middle East, Africa	f	n.n.	quantitative	no	no
Waage et al. 2016[33]	Western Europe, Eastern Europe, Middle East, Africa, South Asia, East Asia	f	n.n.	quantitative	no	no
Grewal et al. 2016[34]	Somalia, Iraq	c	n.n.	quantitative	no	no
Oftedal et al. 2016[35]	various	f/m	n.n.	quantitative	no	no

(Continued)

Table 1. (Continued)

Reference	Characteristics of study participants			Methodology	Intervention (yes/no – not relevant)	User-involvement described (yes/no – not relevant)
	Geographic background	Gender/age group (f=female; m=male; c=child; a=adolescent)	Immigrant status (n.n.=not described –=not relevant)			
Wandel et al. 2016[36]	Somalia	f	n.n	qualitative	no	no
Eggemoen et al. 2016[37]	various	f/m	n.n.	quantitative	no	no
Kinnunen et al. 2016[38]	Western Europe, Eastern Europe, Middle East, Africa, South Asia, East Asia	f	n.n.	quantitative	no	no
Waage et al. 2017[39]	South Asia, Middle East, Africa, East Asia, Eastern Europe	f	n.n	quantitative	no	no
Garnweidner-Holme et al. 2017[40]	various	f	n.n.	qualitative	no	no
Kinnunen et al. 2017[41]	Europe, Middle East, South Asia, East Asia, Africa	f	n.n	quantitative	no	no
Eggemoen et al. 2017[42]	various	f/m	n.n.	quantitative	no	no
Bærug et al. 2018[43]	Western Europe, South Asia, Middle East	f	n.n.	quantitative	no	no
Clark et al. 2018[44]	Somalia	f	n.n	qualitative	no	no
Sletner et al. 2018[45]	Europe, South Asia, East Asia, Middle East/North Africa	f/m	n.n.	quantitative	no	no
Nilsen et al. 2018[46]	various	f	n.n.	quantitative	no	no
Sole et al. 2018[47]	Europe, North America, Latin America/Caribbean, Middle East/ North Africa, Sub-Saharan Africa, Transcaucasia, Central Asia, South Asia, East Asia, Oceania	f	n.n.	quantitative	no	no
Eggemoen et al. 2018[48]	Europe, South Asia, East Asia, Middle East, Sub-Saharan Africa	f	n.n.	quantitative	no	no
Torkildsen et al. 2019[49]	Nordic Countries, Europe, North America, Latin America/ Carribean, Middle East/North Africa, sub-Saharan Africa, Transcaucasia/Central Asia, South Asia, East Asia, Oceania	f	n.n.	quantitative	no	no
Bakken et al. 2019[50]	Western Europe, Eastern Europe, Latin America/Carribean, East Asia, Southeast Asia, Central Asia, South Asia, West Asia, Africa	f	n.n.	quantitative	no	no
Gele et al. 2019[51]	Somalia	f	n.n.	quantitative	no	no
Nilsen et al. 2019[52]	Central Europe, Eastern Europe, Central Asia, Latin America/ Carribean, North Africa/Middle East, South Asia, Southeast Asia, East Asia, Oceania, Sub-Saharan Africa	f	n.n.	quantitative	no	no
Næss- Andresen et al. 2019[53]	Western Europe, South Asia, Middle East, Sub-Saharan Africa, East Asia, Eastern Europe	f	n.n.	quantitative	no	no
Diaz et al. 2019[54]	Pakistan, Vietnam, Morocco, Chile, Poland	f	n.n.	quantitative	no	no
Mæland et al. 2019[55]	Non-western	f	n.n.	quantitative	no	no
Kinnunen et al. 2019[56]	western Europe, Eastern Europe, Middle East, Africa, South Asia, East Asia	f	n.n.	quantitative	no	no
Henriksen et al. 2019[57]	Pakistan, Somalia	f	n.n.	quantitative	yes (RCT protocol)	yes
Vik et al. 2019[58]	various	f	n.n.	quantitative	no	no
Gele et al. 2020[59]	Somalia	f	n.n.	qualitative	no	no
Eskild et al. 2020[60]	Afghanistan, Somalia, Iraq	f	n.n.	quantitative	no	no

(Continued)

Table 1. (Continued)

Reference	Characteristics of study participants			Methodology	Intervention (yes/no – not relevant)	User-involvement described (yes/no – not relevant)
	Geographic background	Gender/age group (f=female; m=male; c=child; a=adolescent)	Immigrant status (n.n.=not described –=not relevant)			
Mental health						
Sagatun et al. 2008[61]	Various	A	n.n.	quantitative	no	no
Johnsen et al. 2009[62]	Middle East, Yugoslavia, Chile	f/m	n.n.	quantitative	no	no
Vaage et al. 2009[63]	Vietnam	c	refugees	quantitative	no	no
Berg 2009[64]	various	f/m	n.n.	quantitative	no	no
Vaage et al. 2010[65]	Vietnam	f/m	refugees	quantitative	no	no
Berg et al. 2010[66]	various	f/m	n.n.	quantitative	no	no
Vaage et al. 2011[67]	Vietnam	f/m	refugees	quantitative	no	no
Richter et al. 2011[68]	various	a	n.n.	quantitative	no	no
Seglem et al. 2011[69]	Afghanistan, Somalia, Sri Lanka, Iraq	c/a	unaccompanied minors	quantitative	no	no
Iversen et al. 2011[70]	various	f/m	n.n.	quantitative	no	no
Erdal et al. 2011[71]	various	f/m	n.n.	quantitative	no	no
Alves et al. 2011[72]	various	a	n.n.	quantitative	no	no
Berg et al. 2011[73]	various	f/m	n.n.	quantitative	no	no
Teodorescu et al. 2012[74]	various	f/m	refugees	quantitative	no	no
Teodorescu et al 2012[75]	various	f/m	refugees	quantitative	no	no
Eide et al. 2013[76]	various	c/a	unaccompanied minors	review	–	–
Stenmark et al. 2013[77]	various	f/m	refugees, asylum seekers	quantitative	yes (RCT)	no
Saheer et al. 2013[78]	Sri-Lanka, Pakistan, Iran, Vietnam, Turkey	f/m	n.n.	quantitative	no	no
Jensen at al. 2014[79]	various	c/a	unaccompanied minors	quantitative	no	no
Jakobsen et al. 2014[80]	Afghanistan, Somalia	a	unaccompanied minors	quantitative	no	no
Berg et al. 2014[81]	various	f/m	n.n.	quantitative	no	no
Abebe et al. 2014[82]	various	f/m	n.n.	review	–	–
Bratt 2015[83]	various	a	n.n.	quantitative	no	no
Opaas et al. 2015[84]	various	f/m	refugees	quantitative	no	no
Oppedal et al. 2015[85]	various	c/a	unaccompanied minors	quantitative	no	no
Tingvold et al. 2015[86]	Vietnam	f/m	refugees	quantitative	no	no
Jensen et al. 2015[87]	various	c/a	unaccompanied minors	quantitative	no	no
Keles et al. 2016[88]	various	c/a	unaccompanied minors	quantitative	no	no
Opaas et al. 2016[89]	Asia, Eastern Europe, Africa, Middle East	f/m	refugees	quantitative	no	no
Straiton et al. 2016[90]	Sweden, Poland, Philippines, Thailand, Pakistan, Russia	f	n.n.	quantitative	no	no

(Continued)

Table 1. (Continued)

Reference	Characteristics of study participants			Methodology	Intervention (yes/no – not relevant)	User-involvement described (yes/no – not relevant)
	Geographic background	Gender/age group (f=female; m=male; c=child; a=adolescent)	Immigrant status (n.n.=not described –=not relevant)			
Myhrvold et al. 2016[91]	various/Norwegian healthcare providers	f/m	undocumented migrants	mixed methods	no	no
Markova et al. 2016[92]	Somalia	f/m	refugees	mixed methods	no	no
Straiton et al. 2017[93]	Iraq, Somalia, Bosnia and Herzegovina, Iran, Kosovo, Afghanistan	f/m	n.n.	quantitative	no	no
Jakobsen et al. 2017[94]	Afghanistan, Somalia	c/a	unaccompanied minors	quantitative	no	no
Berg et al. 2017[95]	various	f/m	n.n.	quantitative	no	no
Puzo et al. 2017[96]	various	f/m	n.n.	quantitative	no	no
Keles et al. 2017[97]	various	c/a	unaccompanied minors	quantitative	no	no
Jakobsen et al. 2017[98]	various	c/a	unaccompanied minors	quantitative	no	no
Abebe et al. 2017[99]	various	f/m	n.n	quantitative	no	no
Finnvold et al. 2018[100]	various	f/m	refugees	quantitative	no	no
Puzo et al. 2018[101]	various	f/m	n.n.	quantitative	no	no
Sagbakken et al. 2018[102]	Norwegian healthcare providers	f/m	–	qualitative	no	no
Keles et al. 2018[103]	various	c	n.n.	quantitative	no	no
Abraham et al. 2018[104]	Eritrea	f	asylum seekers	qualitative	no	no
Puzo et al. 2018[105]	various	f/m	n.n.	quantitative	no	no
Tschirhart et al. 2019[106]	Thailand	f	n.n.	qualitative	no	no
Hjellset et al. 2019[107]	Pakistan	f	n.n.	quantitative	no	no
Jensen et al. 2019[108]	various	c/a	refugees	quantitative	no	no
Øien-Ødegaard et al. 2019[109]	various	f/m	n.n.	quantitative	no	no
Høyvik et al. 2019[110]	various	f/m	refugees	quantitative	no	no
Straiton et al. 2019[111]	various	f/m	n.n.	quantitative	no	no
Cardiovascular diseases and risk factors						
Kolsgaard et al. 2008[112]	Pakistan, Turkey, Tamil region	f/m	n.n.	quantitative	no	no
Sagatun et al. 2008[113]	various	a	n.n.	quantitative	no	no
Kumar et al. 2009[114]	Sri-Lanka, Pakistan, Iran, Vietnam, Turkey	f/m	n.n.	quantitative	no	no
Hussain et al. 2010[115]	Pakistan	f	n.n.	quantitative	no	no
Tennakoon et al. 2010[116]	Sri Lanka	f/m	n.n.	quantitative	no	no
Råberg et al. 2010[117]	South Asia	f/m	n.n.	quantitative	no	no
Råberg Kjøllesdal 2010[118]	Pakistan	f	n.n.	quantitative	no	no
Råberg Kjøllesdal 2011[119]	Pakistan	f	n.n.	quantitative	yes (RCT)	no
Råberg Kjøllesdal 2011[120]	Pakistan	f	n.n.	quantitative	yes (RCT)	no
Tran et al. 2011[121]	Sri-Lanka, Pakistan, Iran, Vietnam, Turkey	f/m	n.n.	quantitative	no	no
Hjellset et al. 2011[122]	Pakistan	f	n.n.	quantitative	no	no
Råberg Kjøllesdal 2011[123]	Pakistan	f	n.n.	quantitative	yes (RCT)	no
Lunde et al. 2012[124]	Pakistan	f	n.n.	quantitative	no	no

(Continued)

Table 1. (Continued)

Reference	Characteristics of study participants			Methodology	Intervention (yes/no – not relevant)	User-involvement described (yes/no – not relevant)
	Geographic background	Gender/age group (f=female; m=male; c=child; a=adolescent)	Immigrant status (n.n.=not described –=not relevant)			
Abdelnoor et al. 2012[125]	various	f/m	n.n.	quantitative	no	no
Jenum et al. 2012[126]	Turkey, Vietnam, Sri Lanka, Pakistan	f/m	n.n.	quantitative	no	no
Andersen et al. 2012[127]	Pakistan	m	n.n.	quantitative	yes (RCT)	yes
Iversen et al. 2013[128]	Iran, Pakistan, Vietnam, Sri Lanka, Turkey	f/m	n.n.	quantitative	no	no
Tennakoon et al. 2013[129]	Sri Lanka	f/m	n.n.	quantitative	no	no
Rabanal et al. 2013[130]	Eastern Europe, Former Yugoslavia, North America, Sub-Saharan Africa, Middle-East, Indian subcontinent, East Asia, North America, South America, South-east Asia	f/m	n.n.	quantitative	no	no
Gele et al. 2013[131]	Somalia	f/m	n.n.	quantitative	no	no
Helland-Kigen et al. 2013[132]	Pakistan	f	n.n.	quantitative	yes (RCT)	no
Telle-Hjellseth et al. 2013[133]	Pakistan	f	n.n.	quantitative	yes (RCT)	no
Tran et al. 2013[134]	various	f/m	n.n.	quantitative	no	no
Wium et al. 2013[135]	Pakistan	m	n.n.	quantitative	no	no
Andersen et al. 2013[136]	Pakistan	m	n.n.	quantitative	yes (RCT)	yes
Tennakoon et al. 2015[137]	Sri Lanka	f/m	n.n.	quantitative	no	no
Andersen et al. 2015[138]	Pakistan	m	n.n.	quantitative	no	no
Rabanal et al. 2015[139]	various	f/m	n.n.	quantitative	no	no
Gele et al. 2016[140]	Somalia	f	n.n.	quantitative	no	no
Rabanal et al. 2017[141]	South Asia	f/m	n.n.	quantitative	no	no
Ahmed et al. 2018[142]	Somalia	f/m	n.n.	quantitative	no	no
Toftemo et al. 2018[143]	Europe, South Asia, Middle East/North Africa	c	n.n.	quantitative	no	no
Abuelmagd et al. 2018[144]	Pakistan	f	n.n.	qualitative	no	no
Tran et al. 2019[145]	Western Europe, Eastern Europe, East Asia, South Asia, East Africa, Middle East/North Africa	f/m	n.n.	quantitative	no	no
Infectious diseases						
Winje et al. 2008[146]	various	f/m	n.n.	quantitative	no	no
Harstad et al. 2009[147]	various	f/m	asylum seekers	quantitative	no	no
Harstad et al. 2009[148]	various	f/m	asylum seekers	quantitative	no	no
Krogh et al. 2010[149]	various	c	n.n.	quantitative	no	no
Harstad et al. 2010[150]	various	f/m	asylum seekers	quantitative	no	no
Harstad et al. 2010[151]	various	f/m	asylum seekers	quantitative	no	no
Bjerke et al. 2010[152]	Pakistan	f/m	n.n.	quantitative	no	no
Sagbakken et al. 2010[153]	Somalia, Ethiopia	f/m	n.n.	qualitative	no	no
Hassan et al. 2013[154]	Pakistan	f/m	n.n.	quantitative	no	no
Pullar et al. 2014[155]	various	f/m	n.n.	quantitative	no	no
Harstad et al. 2014[156]	various	f/m	n.n.	quantitative	yes (non-randomized trial)	yes

(Continued)

Table 1. (Continued)

Reference	Characteristics of study participants			Methodology	Intervention (yes/no – not relevant)	User-involvement described (yes/no – not relevant)
	Geographic background	Gender/age group (f=female; m=male; c=child; a=adolescent)	Immigrant status (n.n.=not described –=not relevant)			
Guzman Herrador et al. 2015[157]	various	f/m	n.n.	quantitative	no	no
Jensenius et al. 2016[158]	various	f/m	n.n.	quantitative	no	no
Haukaas et al. 2017[159]	various	f/m	n.n.	quantitative	no	no
Di Ruscio et al. 2017[160]	various	f/m	n.n.	quantitative	no	no
Asfeldt et al. 2018[161]	various	f/m	asylum seekers	quantitative	no	no
Danielsen et al. 2019[162]	various	f/m	asylum seekers	quantitative	no	no
Festvåg et al. 2019[163]	Western, Non-Western	f/m	n.n.	quantitative	no	no
Winje et al. 2019[164]	Myanmar, Philippines, Somalia, Pakistan, Ethiopia, Afghanistan, Thailand, India, Vietnam, Eritrea, Horn of Africa	f/m	n.n.	quantitative	no	no
Nordstoga et al. 2019[165]	various	f/m	n.n.	qualitative	no	no
Gynaecology						
Gele et al. 2012[166]	Somalia	f	n.n.	qualitative	no	no
Gele et al. 2012[167]	Somalia	f/m	n.n.	quantitative	no	no
Schultz et al. 2013[168]	Somalia, Gambia	f	n.n.	qualitative	no	yes
Gele et al. 2015[169]	Somalia	a	n.n.	qualitative	no	yes
Ziyada et al. 2016[170]	various	f/m	n.n.	quantitative	no	no
Leinonen et al. 2017[171]	various	f	n.n.	quantitative	no	no
Møen et al. 2017[172]	various	f/m	n.n.	quantitative	no	no
Gele et al. 2017[173]	Pakistan, Somalia	f	n.n.	qualitative	no	yes
Johansen 2017[174]	Somalia, Sudan	f/m	n.n.	qualitative	no	no
Johansen 2017[175]	Somalia, Sudan	f/m	n.n.	qualitative	no	no
Mbanya et al. 2018[176]	Somalia	f/m	n.n.	quantitative	no	no
Møen et al. 2018[177]	Norwegian healthcare providers	f	–	qualitative	no	no
Bhargava et al. 2018[178]	High income, Middle income, Low income countries	f	n.n.	quantitative	no	no
Johansen 2019[179]	Somalia	f	n.n.	qualitative	no	yes
Bhargava et al. 2019[180]	various	f	n.n.	qualitative	no	no
Qureshi et al. 2019[181]	Pakistan, Somalia	f	n.n.	quantitative	yes (RCT)	yes
Endocrine diseases						
Meyer et al. 2008[182]	Sri Lanka	f/m	n.n.	quantitative	no	no
Madar et al. 2009[183]	Pakistan, Turkey, Somalia	c	n.n.	quantitative	yes	no
Madar et al. 2009[184]	Pakistan, Turkey, Somalia	f,c	n.n.	quantitative	no	no
Knutsen et al. 2010[185]	various	f/m	n.n.	quantitative	no	no
Eggemoen et al. 2013[186]	Africa, Asia	f/m	n.n.	quantitative	no	no
Knutsen et al. 2014[187]	Middle East, Africa, South Asia	f/m	n.n.	quantitative	yes (RCT)	no
Wium et al. 2014[188]	Pakistan	f/m	n.n.	quantitative	no	no
Rasmussen et al. 2014[189]	Pakistan	c	n.n.	quantitative	no	no
Madar et al. 2014[190]	South Asia, Middle East, Africa	f/m	n.n.	quantitative	yes (RCT)	no
Madar et al. 2015[191]	South Asia, Middle East, Africa	f/m	n.n.	quantitative	yes (RCT)	no
Dzidzonu et al. 2016[192]	various	f/m	n.n.	quantitative	no	no

(Continued)

Table 1. (Continued)

Reference	Characteristics of study participants			Methodology	Intervention (yes/no – not relevant)	User-involvement described (yes/no – not relevant)
	Geographic background	Gender/age group (f=female; m=male; c=child; a=adolescent)	Immigrant status (n.n.=not described –=not relevant)			
Meyer et al. 2017[193]	various	f/m	n.n.	quantitative	no	no
Madar et al. 2017[194]	Africa, Asia, Middle East	c	n.n.	quantitative	no	no
Knutsen et al. 2017[195]	South Asia, Middle East, Africa	f/m	n.n.	quantitative	yes (RCT)	no
Madar et al. 2018[196]	Somalia	f/m	n.n.	quantitative	no	no
Pain						
Log et al. 2011[197]	various	a	n.n.	quantitative	no	no
Borchgrevink 2011[198]	various	f/m	n.n.	quantitative	no	no
Sverre et al. 2014[199]	Pakistan	f	n.n.	qualitative	no	no
Knutsen et al. 2014[200]	Middle East, Africa, South Asia	f/m	n.n.	quantitative	yes (RCT)	no
Teodorescu et al. 2015[201]	various	f/m	refugees	quantitative	no	no
Nortvedt et al. 2015[202]	various	f	n.n.	qualitative	no	no
Nortvedt et al. 2016[203]	Africa, Asia	f	n.n.	qualitative	no	no
Nyen et al. 2018[204]	Non-western	f	n.n.	qualitative	no	no
Hasha et al. 2019[205]	Syria	f/m	refugees	quantitative	yes (protocol for RCTs)	yes
Dental health						
Skeie et al. 2008[206]	various	c	n.n.	quantitative	no	no
Skaret et al. 2008[207]	Non-western	f/m	n.n.	quantitative	no	no
Wigen et al. 2010[208]	Non-western	c	n.n.	quantitative	no	no
Skeie et al. 2010[209]	Non-western	f/m	n.n.	quantitative	no	no
Høyvik et al. 2019[210]	Middle East, Africa	f/m	refugees	quantitative	no	no
Substance abuse						
Amundsen 2012[211]	Non-western	f/m	n.n.	quantitative	no	no
Vedøy 2013[212]	Non-western	f/m	n.n.	quantitative	no	no
Abebe 2015[213]	Europe, United States, Middle East, Africa, Asia	a	n.n.	quantitative	no	no
Skogen et al. 2018[214]	European, non-European	a	n.n.	quantitative	no	no
Neurology						
Smestad et al. 2008[215]	Middle East, Asia, Africa	f/m	n.n.	quantitative	no	no
Berg-Hansen et al. 2013[216]	Non-western	f/m	n.n.	quantitative	no	no
Berg-Hansen et al. 2014[217]	Europe, North America, Oceania/South and Central America, Africa, Asia	f/m	n.n.	quantitative	no	no
Boldingh et al. 2017[218]	Europe, Asia, South America	f/m	n.n.	quantitative	no	no
Oncology						
Latif et al. 2015[219]	Pakistan, Sri Lanka, Somalia	f	n.n.	quantitative	no	no
Thøgersen et al. 2017[220]	Eastern Europe, Middle East, Sub-Saharan Africa, South Asia, East Asia	f/m	n.n.	quantitative	no	no

(Continued)

Table 1. (Continued)

Reference	Characteristics of study participants			Methodology	Intervention (yes/no – not relevant)	User-involvement described (yes/no – not relevant)
	Geographic background	Gender/age group (f=female; m=male; c=child; a=adolescent)	Immigrant status (n.n.=not described –=not relevant)			
Hjerkind et al. 2017[221]	Nordic countries, Western Europe, Eastern Europe, Middle East, South Asia, East Asia, Sub-Saharan Africa, Africa, United States/ Canada, Oceania, South/Central America	f/m	n.n.	quantitative	no	no
Thøgersen et al. 2018[222]	Western, Non-western	f/m	n.n.	quantitative	no	no
Nutrition						
Chen et al. 2018[223]	Somalia	f/m	n.n.	quantitative	no	yes
Henjum et al. 2019[224]	various	f/m	asylum seekers	quantitative	no	yes
Henjum et al. 2019[225]	various	f/m	asylum seekers	quantitative	no	yes
Multimorbitidy						
Diaz et al. 2015[226]	Western countries, Eastern Europe, Non-western countries	f/m	n.n.	quantitative	no	no
Diaz et al. 2015[227]	various	f/m	n.n.	quantitative	no	no
Quality of life						
Hjellset et al. 2011[228]	Pakistan	f	n.n.	quantitative	no	no
Myhrvold et al. 2019[229]	various	f/m	undocumented migrants	mixed methods	no	no
Orthopaedic						
Falch 1983[230]	various	f/m	n.n.	quantitative	no	no
Ortholaryngology						
Amundsen et al. 2017[231]	various	f/m	n.n.	quantitative	no	no
Forensics						
Kvaal et al. 2017[232]	various	c	asylum seekers	quantitative	no	no
Geriatric						
Diaz et al. 2015[233]	various	f/m	n.n.	quantitative	no	no
Mortality						
Syse et al. 2018[234]	various	f/m	n.n.	quantitative	no	no
Vaccination						
Riise et al. 2015[235]	various	c	n.n.	quantitative	no	no
Health status/use of medication						
Strømme et al. 2020[236]	Syria	f/m	refugees	quantitative	no	no
Socio-cultural aspects						
Høye et al. 2008[237]	Norwegian healthcare providers	f/m	–	qualitative	no	no
Varvin et al. 2009[238]	Norwegian healthcare providers	f/m	–	quantitative	no	no
Claussen et al. 2009[239]	Western Europe, Eastern Europe, developing countries	f/m	n.n.	quantitative	no	no
Stige et al. 2010[240]	Tamil region	f/m	n.n.	quantitative	no	no
Tran et al. 2010[241]	Western Europe/North America, South Asia, Middle East/North Africa, East Asia	f/m	n.n.	quantitative	no	no
Høye et al. 2010[242]	Non-western	f/m	n.n.	qualitative	no	no
Hanssen et al. 2010[243]	Norwegian healthcare providers	f/m	–	qualitative	no	no
Madar et al. 2011[244]	Turkey, Somalia, Pakistan	f	n.n.	quantitative	yes (RCT)	no
Kale et al. 2011[245]	Eastern Europe, Middle East, South America, South Asia	f/m	n.n.	qualitative	no	no

(Continued)

Table 1. (Continued)

Reference	Characteristics of study participants			Methodology	Intervention (yes/no – not relevant)	User-involvement described (yes/no – not relevant)
	Geographic background	Gender/age group (f=female; m=male; c=child; a=adolescent)	Immigrant status (n.n.=not described –=not relevant)			
Håkonsen et al. 2011[246]	Pakistan	f/m	n.n.	quantitative	no	no
Småland Goth et al. 2011[247]	various	f/m	n.n.	qualitative	no	no
Guribye 2011[248]	Tamil region	f/m	refugees	qualitative	no	no
Guribye et al. 2011[249]	Tamil region	f/m	refugees	qualitative	no	no
Håkonsen et al. 2012[250]	Pakistan	f/m	n.n.	quantitative	no	no
Claussen et al. 2012[251]	Nordic countries, Western Europe, Eastern Europe, Middle East/ North Africa, Sub-Saharan Africa, Asia, North America, Central South America, Oceania	f/m	n.n.	quantitative	no	no
Alnaes 2012[252]	Non-western	f/m	n.n.	qualitative	no	no
Håkonsen et al. 2014[253]	Norwegian healthcare providers	f/m	–	qualitative	no	no
Debesay et al. 2014[254]	Norwegian healthcare providers	f/m	–	qualitative	no	no
Debesay et al. 2014[255]	Norwegian healthcare providers	f/m	–	qualitative	no	no
Alpers et al. 2014[256]	Norwegian healthcare providers	f/m	–	mixed methods	no	yes
Elstad et al. 2015[257]	various	f/m	n.n.	quantitative	no	no
Holmberg Fagerlund et al. 2016[258]	Norwegian healthcare providers	f/m	–	qualitative	no	no
Gimeno-Feliu et al. 2016[259]	various	f/m	n.n.	quantitative	no	no
Gele et al. 2016[260]	Somalia	f	n.n.	quantitative	no	no
Straiton et al. 2017[261]	Philippines	f	n.n.	qualitative	no	no
Straiton et al. 2017[262]	Thailand, Philippines	f	n.n.	qualitative	no	no
Hjörleifsson et al. 2018[263]	Norwegian healthcare providers	f/m	–	qualitative	no	no
Nosrati et al. 2018[264]	various	f/m	n.n.	quantitative	no	no
Lillehagen et al. 2018[265]	various	f/m	n.n.	quantitative	no	no
Alpers 2018[266]	Asia, Africa	f/m	n.n.	qualitative	no	no
Abuelmagd et al. 2019[267]	Kurdistan	f/m	n.n.	qualitative	no	no
Leirbakk et al. 2019[268]	various	f	n.n.	quantitative	yes (RCT protocol)	yes
Arora et al. 2019[269]	Pakistan	f	n.n.	qualitative	no	no
Ziyada et al. 2020[270]	Sudan, Somalia	f	n.n.	qualitative	no	no
Oppedal et al. 2020[271]	various	f/m	n.n.	quantitative	no	no
Martiny et al. 2020[272]	various	a	n.n.	quantitative	no	no
Use of healthcare services						
Lien et al. 2008[273]	Non-western	f/m	n.n.	quantitative	no	no
Ayazi et al. 2008[274]	Western, Non-western	f/m	n.n.	quantitative	no	no
Sandvik et al. 2012[275]	Poland, Sweden, Germany, Pakistan, Iraq	f/m	n.n.	quantitative	no	no
Diaz et al. 2014[276]	High income countries, other income countries	f/m	n.n.	quantitative	no	no
Diaz et al. 2014[277]	High income, Middle income, Low income countries	f/m	n.n.	quantitative	no	no

(Continued)

Table 1. (Continued)

Reference	Characteristics of study participants			Methodology	Intervention (yes/no – not relevant)	User-involvement described (yes/no – not relevant)
	Geographic background	Gender/age group (f=female; m=male; c=child; a=adolescent)	Immigrant status (n.n.=not described –=not relevant)			
Goth et al. 2014[278]	various	f/m	n.n.	quantitative	no	no
Straiton et al. 2014[279]	Poland, Sweden, Germany, Pakistan, Iraq	f/m	n.n.	quantitative	no	no
Diaz et al. 2015[280]	Low Income, Lower-middle income, Upper- middle income, High income countries	f/m	n.n.	quantitative	no	no
Ruud et al. 2015[281]	Sweden, Somalia, Pakistan, Poland	f/m	n.n.	quantitative	no	no
Gele et al. 2015[282]	Somalia	f	n.n.	qualitative	no	no
Fadnes et al. 2016[283]	various	c	n.n.	quantitative	no	no
Ruud et al. 2016[284]	Nordic countries, Western Europe/North America/Oceania, Eastern Europe, Asia, Africa, Latin America	f/m	n.n.	quantitative	no	no
Elstad 2016[285]	Western countries, Eastern Europe, Africa, West and South Asia, Asia, Latin America	f/m	n.n.	quantitative	no	no
Aarseth et al. 2016[286]	Norwegian healthcare providers	f/m	undocumented migrants	quantitative	no	no
Czapka et al. 2016[287]	Poland	f/m	n.n.	qualitative	no	no
Tatara et al. 2016[288]	Pakistan	f/m	n.n.	quantitative	no	no
Ruud et al. 2017[289]	various	f/m	n.n.	quantitative	no	no
Fadnes et al. 2017[290]	various	c	n.n.	quantitative	no	no
Diaz et al. 2017[291]	Sub-Saharan Africa	f/m	n.n.	quantitative	no	no
Tatara et al. 2017[292]	Pakistan	f/m	n.n.	quantitative	no	no
Straiton et al. 2018[293]	Philippines	f	n.n.	qualitative	no	no
Abebe et al. 2018[294]	Western Europe, Eastern Europe, Africa, Asia, Latin America	f/m	n.n.	quantitative	no	no
Finnvold 2018[295]	Sweden, Poland, Vietnam, Russia, Somalia, Sri Lanka, Turkey, Bosnia and Herzegovina, Iran, Iraq, Afghanistan	f/m	n.n.	quantitative	no	no
Sagbakken et al. 2018[296]	various/Norwegian healthcare personnel	f/m	n.n.	qualitative	no	no
Straiton et al. 2019[297]	Western Europe, Eastern Europe, Middle East/North Africa, Sub-Saharan Africa, South Asia, East/Southeast Asia	f	n.n.	quantitative	no	no
Schein et al. 2019[298]	Ethiopia	f/m	refugees and asylum seekers	qualitative	no	no
Tschirhart et al. 2019[299]	Thailand	f	n.n.	qualitative	no	no
Czapka et al. 2019[300]	Poland	f/m	n.n.	qualitative	no	no
Mbanya et al. 2019[301]	Sub-Saharan Africa	f/m	n.n.	qualitative	no	no
Arfa et al. 2020[302]	Non-western	f/m	n.n.	qualitative	no	no
Mbanya et al. 2020[303]	Sub-Saharan Africa	f	n.n.	qualitative	no	no

[1]Bjerke SEY, Vangen S, Nordhagen R, et al. Postpartum depression among Pakistani women in Norway: prevalence and risk factors. *J Matern-Fetal Neonatal Med* 2008;21:889–894.
[2]Vikanes A, Grjibovski AM, Vangen S, et al. Length of residence and risk of developing hyperemesis gravidarum among first generation immigrants to Norway. *Eur J Public Health* 2008;18:460–465.
[3]Vangen S, Eskild A and Forsen L. Termination of pregnancy according to immigration status: a population-based registry linkage study. *Bjog* 2008;115:1309–1315.
[4]Grjibovski AM, Magnus P and Stoltenberg C. Decrease in consanguinity among parents of children born in Norway to women of Pakistani origin: a registry-based study. *Scand J Public Health* 2009;37:232–238.
[5]Jenum AK, Sletner L, Voldner N, et al. The STORK Groruddalen research programme: a population-based cohort study of gestational diabetes, physical activity, and obesity in pregnancy in a multiethnic population. Rationale, methods, study population, and participation rates. *Scand J Public Health* 2010;38:60–70.
[6]Singh N, Pripp AH, Brekke T, et al. Different sex ratios of children born to Indian and Pakistani immigrants in Norway. *BMC Pregnancy Childbirth* 2010;10:40.

Table 1. (Continued)

[7]Bjerke SE, Vangen S, Holter E, et al. Infectious immune status in an obstetric population of Pakistani immigrants in Norway. *Scand J Public Health* 2011;39:464–470.
[8]Bø K, Pauck Øglund G, Sletner L, et al. The prevalence of urinary incontinence in pregnancy among a multi-ethnic population resident in Norway. *Bjog* 2012;119:1354–1360.
[9]Lyberg A, Viken B, Haruna M, et al. Diversity and challenges in the management of maternity care for migrant women. *J Nurse Manag* 2012;20:287–295.
[10]Mørkrid K, Jenum AK, Sletner L, et al. Failure to increase insulin secretory capacity during pregnancy-induced insulin resistance is associated with ethnicity and gestational diabetes. *Eur J Endocrinol* 2012;167:579–588.
[11]Sachse D, Sletner L, Mørkrid K, et al. Metabolic changes in urine during and after pregnancy in a large, multiethnic population-based cohort study of gestational diabetes. *PloS One* 2012;7:e52399.
[12]Tønnessen M, Aalandslid V and Skjerpen T. Changing trend? Sex ratios of children born to Indian immigrants in Norway revisited. *BMC Pregnancy Childbirth* 2013;13:170.
[13]Brekke I, Berg JE, Sletner L, et al. Doctor-certified sickness absence in first and second trimesters of pregnancy among native and immigrant women in Norway. *Scand J Public Health* 2013;41:166–173.
[14]Garnweidner LM, Sverre Pettersen K and Mosdøl A. Experiences with nutrition-related information during antenatal care of pregnant women of different ethnic backgrounds residing in the area of Oslo, Norway. *Midwifery* 2013;29:e130–e137.
[15]Naimy Z, Grytten J, Monkerud L, et al. Perinatal mortality in non-western migrants in Norway as compared to their countries of birth and to Norwegian women. *BMC Public Health* 2013;13:37.
[16]Vikanes AV, Støer NC, Gunnes N, et al. *Helicobacter pylori* infection and severe hyperemesis gravidarum among immigrant women in Norway: a case-control study. *Eur J Obstat Gynecol Reprod Biol* 2013;167:41–46.
[17]Sletner L, Nakstad B, Yajnik CS, et al. Ethnic differences in neonatal body composition in a multi-ethnic population and the impact of parental factors: a population-based cohort study. *PloS One* 2013;8:e73058.
[18]Sletner L, Jenum AK, Mørkrid K, et al. Maternal life course socio-economic position and offspring body composition at birth in a multi-ethnic population. *Paediatr Perinat Epidemiol* 2014;28:445–454.
[19]Berntsen S, Richardsen KR, Mørkrid K, et al. Objectively recorded physical activity in early pregnancy: a multiethnic population-based study. *Scand J Med Sci Sports* 2014;24:594-601.
[20]Sørbye IK, Stoltenberg C, Sundby J, et al. Stillbirth and infant death among generations of Pakistani immigrant descent: a population-based study. *Acta Obstet Gynecol Scand* 2014;93:168–174.
[21]Sørbye IK, Daltveit AK, Sundby J, et al. Preterm subtypes by immigrants' length of residence in Norway: a population-based study. *BMC Pregnancy Childbirth* 2014;14:239.
[22]Omland G, Ruths S and Diaz E. Use of hormonal contraceptives among immigrant and native women in Norway: data from the Norwegian Prescription Database. *Bjog* 2014;121:1221–1228.
[23]Shakeel N, Eberhard-Gran M, Sletner L, et al. A prospective cohort study of depression in pregnancy, prevalence and risk factors in a multi-ethnic population. *BMC Pregnancy Childbirth* 2015;15:5.
[24]Sørbye IK, Daltveit AK, Sundby J, et al. Caesarean section by immigrants' length of residence in Norway: a population-based study. *Eur J Public Health* 2015;25:78–84.
[25]Naimy Z, Grytten J, Monkerud L, et al. The prevalence of pre-eclampsia in migrant relative to native Norwegian women: a population-based study. *BJOG* 2015;122:859–865.
[26]Viken B, Lyberg A and Severinsson E. Maternal health coping strategies of migrant women in Norway. *Nurse Res Pract* 2015;2015:878040.
[27]Bakken KS, Skjeldal OH and Stray-Pedersen B. Higher risk for adverse obstetric outcomes among immigrants of African and Asian descent: a comparison study at a low-risk maternity hospital in Norway. *Birth* 2015;42:132–140.
[28]Bakken KS, Skjeldal OH and Stray-Pedersen B. Immigrants from conflict-zone countries: an observational comparison study of obstetric outcomes in a low-risk maternity ward in Norway. *BMC Pregnancy Childbirth* 2015;15:163.
[29]Sletner L, Rasmussen S, Jenum AK, et al. Ethnic differences in fetal size and growth in a multi-ethnic population. *Early Hum Dev* 2015;91:547–554.
[30]Richardsen KR, Mdala I, Berntsen S, et al. Objectively recorded physical activity in pregnancy and postpartum in a multi-ethnic cohort: association with access to recreational areas in the neighbourhood. *J Behav Nutr Phys Act* 2016;13:78.
[31]Sommer C, Gulseth HL, Jenum AK, et al. Soluble leptin receptor and risk of gestational diabetes in a multiethnic population: a prospective cohort study. *J Clin Endocrinol Metab* 2016;101:4070–4075.
[32]Waage CW, Mdala I, Jenum AK, et al. Ethnic differences in blood pressure from early pregnancy to postpartum: a Norwegian cohort study. *J Hypertens* 2016;34:1151–1159.
[33]Waage CW, Falk RS, Sommer C, et al. Ethnic differences in postpartum weight retention: a Norwegian cohort study. *Bjog* 2016;123:699–708.
[34]Grewal NK, Andersen LF, Sellen D, et al. Breast-feeding and complementary feeding practices in the first 6 months of life among Norwegian-Somali and Norwegian-Iraqi infants: the InnBaKost survey. *Public Health Nutr* 2016;19:703–715.
[35]Oftedal AM, Busterud K, Irgens LM, et al. Socio-economic risk factors for preterm birth in Norway 1999-2009. *Scand J Public Health* 2016;44:587–592.
[36]Wandel M, Terragni L, Nguyen C, et al. Breastfeeding among Somali mothers living in Norway: Attitudes, practices and challenges. *Women Birth* 2016;29:487–493.
[37]Eggemoen ÅR, Falk RS, Knutsen KV, et al. Vitamin D deficiency and supplementation in pregnancy in a multiethnic population-based cohort. *BMC Pregnancy Childbirth* 2016;16:7.
[38]Kinnunen TI, Waage CW, Sommer C, et al. Ethnic differences in gestational weight gain: a population-based cohort study in Norway. *Matern Child Health J* 2016;20:1485–1496.
[39]Waage C, Jenum AK, Mdala I, et al. Associations between gestational diabetes mellitus and elevated HbA(1c) early postpartum in a multi-ethnic population. *Prim Care Diabetes* 2017;11:132–139.
[40]Garnweidner-Holme LM, Lukasse M, Solheim M, et al. Talking about intimate partner violence in multi-cultural antenatal care: a qualitative study of pregnant women's advice for better communication in South-East Norway. *BMC Pregnancy Childbirth* 2017;17:123.
[41]Kinnunen TI, Sletner L, Sommer C, et al. Ethnic differences in folic acid supplement use in a population-based cohort of pregnant women in Norway. *BMC Pregnancy Childbirth* 2017;17:143.
[42]Eggemoen Å R, Jenum AK, Mdala I, et al. Vitamin D levels during pregnancy and associations with birth weight and body composition of the newborn: a longitudinal multiethnic population-based study. *Br J Nutr* 2017;117:985–993.
[43]Bærug A, Sletner L, Laake P, et al. Recent gestational diabetes was associated with mothers stopping predominant breastfeeding earlier in a multi-ethnic population. *Acta Paediatr* 2018;107:1028–1035.
[44]Clark CL, Glavin K, Missal BE, et al. Is there a common experience? Somali new mothers' childbirth experiences in Norway and the United States. *Public Health Nurs* 2018;35:184–191.
[45]Sletner L, Kiserud T, Vangen S, et al. Effects of applying universal fetal growth standards in a Scandinavian multi-ethnic population. *Acta Obstet Gynecol Scand* 2018;97:168–179.
[46]Nilsen RM, Vik ES, Rasmussen SA, et al. Preeclampsia by maternal reasons for immigration: a population-based study. *BMC Pregnancy Childbirth* 2018;18:423.
[47]Sole KB, Staff AC and Laine K. The association of maternal country of birth and education with hypertensive disorders of pregnancy: a population-based study of 960 516 deliveries in Norway. *Acta Obstet Gynecol Scand* 2018;97:1237–1247.
[48]Eggemoen Å R, Waage CW, Sletner L, et al. Vitamin D, gestational diabetes, and measures of glucose metabolism in a population-based multiethnic cohort. *J Diabetes Res* 2018;2018:8939235.
[49]Torkildsen SE, Svendsen H, Räisänen S, et al. Country of birth and county of residence and association with overweight and obesity-a population-based study of 219,555 pregnancies in Norway. *J Public Health (Oxford)* 2019;41:e290–e299.

Table 1. (Continued)

[50]Bakken KS and Stray-Pedersen B. Emergency cesarean section among women in Robson groups one and three: A comparison study of immigrant and Norwegian women giving birth in a low-risk maternity hospital in Norway. *Health Care Women Int* 2019;40:761–775.
[51]Gele AA, Musse FK and Qureshi S. Unmet needs for contraception: a comparative study among Somali immigrant women in Oslo and their original population in Mogadishu, Somalia. *PloS One* 2019;14:e0220783.
[52]Nilsen RM, Daltveit AK, Iversen MM, et al. Preconception folic acid supplement use in immigrant women (1999–2016). *Nutrients* 2019;11.
[53]Næss-Andresen M-L, Eggemoen ÅR, Berg JP, et al. Serum ferritin, soluble transferrin receptor, and total body iron for the detection of iron deficiency in early pregnancy: a multiethnic population-based study with low use of iron supplements. *Am J Clin Nutr* 2019;109:566–575.
[54]Diaz E, Omland G, Hannestad Y, et al. Use of hormonal contraceptives among immigrant women and their daughters in Norway: data from the Norwegian Prescription Database. *Acta Obstet Gynecol Scand* 2019;98:232–239.
[55]Mæland KS, Sande RK and Bing-Jonsson PC. Risk for delivery complications in Robson Group 1 for non-Western women in Norway compared with ethnic Norwegian women – a population-based observational cohort study. *Sex Reproduct Healthc* 2019;20:42–45.
[56]Kinnunen T, Richardsen KR, Sletner L, et al. Ethnic differences in body mass index trajectories from 18 years to postpartum in a population-based cohort of pregnant women in Norway. *BMJ Open* 2019;9.
[57]Henriksen L, Flaathen EM, Angelshaug J, et al. The Safe Pregnancy study – promoting safety behaviours in antenatal care among Norwegian, Pakistani and Somali pregnant women: a study protocol for a randomized controlled trial. *BMC Public Health* 2019;19:724.
[58]Vik ES, Aasheim V, Schytt E, et al. Stillbirth in relation to maternal country of birth and other migration related factors: a population-based study in Norway. *BMC Pregnancy Childbirth* 2019;19:5.
[59]Gele AA, Musse FK, Shrestha M, et al. Barriers and facilitators to contraceptive use among Somali immigrant women in Oslo: a qualitative study. *PloS One* 2020;15:e0229916.
[60]Eskild A, Sommerfelt S, Skau I, et al. Offspring birthweight and placental weight in immigrant women from conflict-zone countries; does length of residence in the host country matter? A population study in Norway. *Acta Obstet Gynecol Scand* 2020;99:615–622.
[61]Sagatun A, Lien L, Søgaard AJ, et al. Ethnic Norwegian and ethnic minority adolescents in Oslo, Norway. A longitudinal study comparing changes in mental health. *Soc Psychiatry Psychiatr Epidemiol* 2008;43:87–95.
[62]Johnsen GE and Asbjørnsen AE. Verbal learning and memory impairments in posttraumatic stress disorder: the role of encoding strategies. *Psychiatry Res* 2009;165:68–77.
[63]Vaage AB, Tingvold L, Hauff E, et al. Better mental health in children of Vietnamese refugees compared with their Norwegian peers – a matter of cultural difference? *Psychiatry Ment Health* 2009;3:34.
[64]Berg JE. The level of non-Western immigrants' use of acute psychiatric care compared with ethnic Norwegians over an 8-year period. *Nord J Psychiatry* 2009;63:217–222.
[65]Vaage AB, Thomsen PH, Silove D, et al. Long-term mental health of Vietnamese refugees in the aftermath of trauma. *Br J Psychiatry* 2010;196:122–125.
[66]Berg AO, Melle I, Torjesen PA, et al. A cross-sectional study of vitamin D deficiency among immigrants and Norwegians with psychosis compared to the general population. *J Clin Psychiatry* 2010;71:1598–1604.
[67]Vaage AB, Thomsen PH, Rousseau C, et al. Paternal predictors of the mental health of children of Vietnamese refugees. *Psychiatry Ment Health* 2011;5:2.
[68]Richter J, Sagatun Å, Heyerdahl S, et al. The Strengths and Difficulties Questionnaire (SDQ) – self-report. An analysis of its structure in a multiethnic urban adolescent sample. *J Child Psychol Psychiatry* 2011;52:1002–1011.
[69]Seglem KB, Oppedal B and Raeder S. Predictors of depressive symptoms among resettled unaccompanied refugee minors. *Scand J Psychol* 2011;52:457–464.
[70]Iversen VC, Berg JE, Småvik R, et al. Clinical differences between immigrants voluntarily and involuntarily admitted to acute psychiatric units: a 3-year prospective study. *J Psychiatr Ment Health Nurs* 2011;18:671–676.
[71]Erdal K, Singh N and Tardif A. Attitudes about depression and its treatment among mental health professionals, lay persons and immigrants and refugees in Norway. *J Affect Disord* 2011;133:481–488.
[72]Alves DE, Roysamb E, Oppedal B, et al. Emotional problems in preadolescents in Norway: the role of gender, ethnic minority status, and home- and school-related hassles. *J Mental Health* 2011;5:37.
[73]Berg AO, Melle I, Rossberg JI, et al. Perceived discrimination is associated with severity of positive and depression/anxiety symptoms in immigrants with psychosis: a cross-sectional study. *BMC Psychiatry* 2011;11:77.
[74]Teodorescu DS, Heir T, Hauff E, et al. Mental health problems and post-migration stress among multi-traumatized refugees attending outpatient clinics upon resettlement to Norway. *Scand J Psychol* 2012;53:316–332.
[75]Teodorescu DS, Siqveland J, Heir T, et al. Posttraumatic growth, depressive symptoms, posttraumatic stress symptoms, post-migration stressors and quality of life in multi-traumatized psychiatric outpatients with a refugee background in Norway. *Health Qual Life Outcomes* 2012;10:84.
[76]Eide K and Hjern A. Unaccompanied refugee children – vulnerability and agency. *Acta Paediatrica* 2013;102:666–668.
[77]Stenmark H, Catani C, Neuner F, et al. Treating PTSD in refugees and asylum seekers within the general health care system. A randomized controlled multicenter study. *Behav Res Ther* 2013;51:641–647.
[78]Saheer TB, Lien L, Hauff E, et al. Ethnic differences in seasonal affective disorder and associated factors among five immigrant groups in Norway. *J Affect Disord* 2013;151:237–242.
[79]Jensen TK, Skårdalsmo EMB and Fjermestad KW. Development of mental health problems – a follow-up study of unaccompanied refugee minors. *J Mental Health* 2014;8:29.
[80]Jakobsen M, Demott MA and Heir T. Prevalence of psychiatric disorders among unaccompanied asylum-seeking adolescents in Norway. *Clin Pract Epidemiol Ment Health* 2014;10:53–58.
[81]Berg AO, Aas M, Larsson S, et al. Childhood trauma mediates the association between ethnic minority status and more severe hallucinations in psychotic disorder. *Psychol Med* 2014;45:133–142.
[82]Abebe DS, Lien L and Hjelde KH. What we know and don't know about mental health problems among immigrants in Norway. *J Immigr Minority Health* 2014;16:60–67.
[83]Bratt C. One of few or one of many: social identification and psychological well-being among minority youth. *Br J Soc Psychol* 2015;54:671–694.
[84]Opaas M and Varvin S. Relationships of childhood adverse experiences with mental health and quality of life at treatment start for adult refugees traumatized by pre-flight experiences of war and human rights violations. *J Nerv Ment Dis* 2015;203:684–695.
[85]Oppedal B and Idsoe T. The role of social support in the acculturation and mental health of unaccompanied minor asylum seekers. *Scand J Psychol* 2015;56:203–211.
[86]Tingvold L, Vaage AB, Allen J, et al. Predictors of acculturative hassles among Vietnamese refugees in Norway: results from a long-term longitudinal study. *Transcult Psychiatry* 2015;52:700–714.
[87]Jensen TK, Fjermestad KW, Granly L, et al. Stressful life experiences and mental health problems among unaccompanied asylum-seeking children. *Clin Child Psychol Psychiatry* 2015;20:106–116.
[88]Keles S, Friborg O, Idsøe T, et al. Depression among unaccompanied minor refugees: the relative contribution of general and acculturation-specific daily hassles. *Ethnicity Health* 2016;21:300–317.
[89]Opaas M, Hartmann E, Wentzel-Larsen T, et al. Relationship of pretreatment Rorschach factors to symptoms, quality of life, and real-life functioning in a 3-year follow-up of traumatized refugee patients. *J Pers Assess* 2016;98:247–260.
[90]Straiton ML, Powell K, Reneflot A, et al. Managing mental health problems among immigrant women attending primary health care services. *Health Care Women Int* 2016;37:118–139.
[91]Myhrvold T and Småstuen M. The mental health care needs of undocumented migrants: an exploratory analysis of psychological distress and living conditions among undocumented migrants in Norway. *J Clin Nurse* 2016;26.

Table 1. (Continued)

[92]Markova V and Sandal GM. Lay explanatory models of depression and preferred coping strategies among Somali refugees in Norway. A mixed-method study. *Front Psychol* 2016;7.

[93]Straiton ML, Reneflot A and Diaz E. Mental health of refugees and non-refugees from war-conflict countries: data from primary healthcare services and the Norwegian Prescription Database. *J Immigr Minority Health* 2017;19:582–589.

[94]Jakobsen M, Meyer DeMott MA, et al. Validity of screening for psychiatric disorders in unaccompanied minor asylum seekers: use of computer-based assessment. *Transcult Psychiatry* 2017;54:611–625.

[95]Berg AO, Melle I, Zuber V, et al. Modelling difficulties in abstract thinking in psychosis: the importance of socio-developmental background. *Cogn Neuropsychiatry* 2017;22:39–52.

[96]Puzo Q, Mehlum L and Qin P. Suicide among immigrant population in Norway: a national register-based study. *Acta Psychiatr Scand* 2017;135:584–592.

[97]Keles S, Idsøe T, Friborg O, et al. The longitudinal relation between daily hassles and depressive symptoms among unaccompanied refugees in Norway. *J Abnorm Child Psychol* 2017;45:1413–1427.

[98]Jakobsen M, Meyer DeMott MA, et al. The impact of the asylum process on mental health: a longitudinal study of unaccompanied refugee minors in Norway. *BMJ Open* 2017;7:e015157.

[99]Abebe DS, Lien L and Elstad JI. Immigrants' utilization of specialist mental healthcare according to age, country of origin, and migration history: a nation-wide register study in Norway. *Soc Psychiatry Psychiatr Epidemiol* 2017;52:679–687.

[100]Finnvold JE and Ugreninov E. Refugees' admission to mental health institutions in Norway: is there an ethnic density effect? *Soc Sci Med* 2018;209:43–50.

[101]Puzo Q, Mehlum L and Qin P. Socio-economic status and risk for suicide by immigration background in Norway: A register-based national study. *J Psychiatr Res* 2018;100:99–106.

[102]Sagbakken M, Spilker RS and Ingebretsen R. Dementia and migration: family care patterns merging with public care services. *Qual Health Res* 2018;28:16–29.

[103]Keles S, Olseth AR, Idsøe T, et al. The longitudinal association between internalizing symptoms and academic achievement among immigrant and non-immigrant children in Norway. *Scand J Psychol* 2018;59:392–406.

[104]Abraham R, Lien L and Hanssen I. Coping, resilience and posttraumatic growth among Eritrean female refugees living in Norwegian asylum reception centres: a qualitative study. *Int J Soc Psychiatry* 2018;64:359–366.

[105]Puzo Q, Mehlum L and Qin P. Rates and characteristics of suicide by immigration background in Norway. *PloS One* 2018;13:e0205035.

[106]Tschirhart N, Straiton M, Ottersen T, et al. 'Living like I am in Thailand': stress and coping strategies among Thai migrant masseuses in Oslo, Norway. *BMC Women's Health* 2019;19:139.

[107]Hjellset VT and Ihlebæk C. Bidimensional acculturation and psychological distress in Pakistani immigrant women in Norway: a cross-sectional study. *J Immigr Minority Health* 2019;21:508–514.

[108]Jensen TK, Skar A-MS, Andersson ES, et al. Long-term mental health in unaccompanied refugee minors: pre- and post-flight predictors. *Eur Child Adolesc Psychiatry* 2019;28:1671–1682.

[109]Øien-Ødegaard C, Reneflot A and Hauge LJ. Use of primary healthcare services prior to suicide in Norway: a descriptive comparison of immigrants and the majority population. *BMC Health Serv Res* 2019;19:508.

[110]Høyvik AC, Lie B and Willumsen T. Dental anxiety in relation to torture experiences and symptoms of post-traumatic stress disorder. *Eur J Oral Sci* 2019;127:65–71.

[111]Straiton ML, Aambø AK and Johansen R. Perceived discrimination, health and mental health among immigrants in Norway: the role of moderating factors. *BMC Public Health* 2019;19:325.

[112]Kolsgaard ML, Andersen LF, Tonstad S, et al. Ethnic differences in metabolic syndrome among overweight and obese children and adolescents: the Oslo Adiposity Intervention Study. *Acta Paediatr* 2008;97:1557–1563.

[113]Sagatun A, Kolle E, Anderssen SA, et al. Three-year follow-up of physical activity in Norwegian youth from two ethnic groups: associations with socio-demographic factors. *BMC Public Health* 2008;8:419.

[114]Kumar BN, Selmer R, Lindman AS, Tverdal A, Falster K and Meyer HE. Ethnic differences in SCORE cardiovascular risk in Oslo, Norway. *Eur J Cardiovasc Prev Rehabil* 2009;16:229–234.

[115]Hussain A, Bjørge B, Hjellset VT, et al. Body size perceptions among Pakistani women in Norway participating in a controlled trial to prevent deterioration of glucose tolerance. *Ethn Health* 2010;15:237–251.

[116]Tennakoon SUB, Kumar BN, Nugegoda DB, et al. Comparison of cardiovascular risk factors between Sri Lankans living in Kandy and Oslo. *BMC Public Health* 2010;10:654.

[117]Råberg M, Kumar B, Holmboe-Ottesen G, et al. Overweight and weight dissatisfaction related to socio-economic position, integration and dietary indicators among south Asian immigrants in Oslo. *Public Health Nutr* 2010;13:695–703.

[118]Råberg Kjøllesdal MK, Telle Hjellset V, Bjørge B, et al. Barriers to healthy eating among Norwegian-Pakistani women participating in a culturally adapted intervention. *Scand J Public Health* 2010;38:52–59.

[119]Råberg Kjøllesdal MK, Hjellset VT, Bjørge B, et al. Perceptions of risk factors for diabetes among Norwegian-Pakistani women participating in a culturally adapted intervention. *Ethn Health* 2011;16:279–297.

[120]Råberg Kjøllesdal MK, Hjellset VT, Bjørge B, et al. Food perceptions in terms of health among Norwegian-Pakistani women participating in a culturally adapted intervention. *Int J Public Health* 2011;56:475–483.

[121]Tran AT, Straand J, Diep LM, et al. Cardiovascular disease by diabetes status in five ethnic minority groups compared to ethnic Norwegians. *BMC Public Health* 2011;11:554.

[122]Hjellset VT, Bjørge B, Eriksen HR, et al. Risk factors for type 2 diabetes among female Pakistani immigrants: the InvaDiab-DEPLAN study on Pakistani immigrant women living in Oslo, Norway. *J Immigr Minority Health* 2011;13:101–110.

[123]Råberg Kjøllesdal MK, Hjellset VT, Bjørge B, et al. Intention to change dietary habits, and weight loss among Norwegian-Pakistani women participating in a culturally adapted intervention. *J Immigr Minority Health* 2011;13:1150–1158.

[124]Lunde MS, Hjellset VT and Høstmark AT. Slow post meal walking reduces the blood glucose response: an exploratory study in female Pakistani immigrants. *J Immigr Minority Health* 2012;14:816–822.

[125]Abdelnoor M, Eritsland J, Brunborg C, et al. Ethnicity and acute myocardial infarction: risk profile at presentation, access to hospital management, and outcome in Norway. *Vasc Health Risk Manag* 2012;8:505–515.

[126]Jenum AK, Diep LM, Holmboe-Ottesen G, et al. Diabetes susceptibility in ethnic minority groups from Turkey, Vietnam, Sri Lanka and Pakistan compared with Norwegians – the association with adiposity is strongest for ethnic minority women. *BMC Public Health* 2012;12:150.

[127]Andersen E, Høstmark AT and Anderssen SA. Effect of a physical activity intervention on the metabolic syndrome in Pakistani immigrant men: a randomized controlled trial. *J Immigr Minority Health* 2012;14:738–746.

[128]Iversen T, Ma CT and Meyer HE. Immigrants' acculturation and changes in body mass index. *Econ Hum Biol* 2013;11:1–7.

[129]Tennakoon SUB, Kumar BN, Selmer R, et al. Differences in predicted cardiovascular risk in Sinhalese and Tamils in Sri Lanka compared with Sri Lankans in Norway. *Asia-Pac J Public Health* 2013;25:452–462.

[130]Rabanal KS, Lindman AS, Selmer RM, et al. Ethnic differences in risk factors and total risk of cardiovascular disease based on the Norwegian CONOR study. *Eur J Prev Cardiol* 2013;20:1013–1021.

[131]Gele AA and Mbalilaki AJ. Overweight and obesity among African immigrants in Oslo. *BMC Research Notes* 2013;6:119.

[132]Helland-Kigen KM, Råberg Kjøllesdal MK, Hjellset VT, et al. Maintenance of changes in food intake and motivation for healthy eating among Norwegian-Pakistani women participating in a culturally adapted intervention. *Public Health Nutr* 2013;16:113–122.

[133]Telle-Hjellset V, Råberg Kjøllesdal MK, Bjørge B, et al. The InnvaDiab-DE-PLAN study: a randomised controlled trial with a culturally adapted education programme improved the risk profile for type 2 diabetes in Pakistani immigrant women. *Br J Nutr* 2013;109:529–538.

Table 1. (Continued)

[134]Tran AT, Straand J, Dalen I, et al. Pharmacological primary and secondary cardiovascular prevention among diabetic patients in a multiethnic general practice population: still room for improvements. *BMC Health Serv Res* 2013;13:182.
[135]Wium C, Aasheim ET, Ueland T, et al. Differences in insulin sensitivity, lipid metabolism and inflammation between young adult Pakistani and Norwegian patients with type 2 diabetes: a cross sectional study. *BMC Endocrine Disorders* 2013;13:49.
[136]Andersen E, Høstmark AT, Holme I, et al. Intervention effects on physical activity and insulin levels in men of Pakistani origin living in Oslo: a randomised controlled trial. *J Immigr Minority Health* 2013;15:101–110.
[137]Tennakoon SU, Kumar BN and Meyer HE. Differences in selected lifestyle risk factors for cardiovascular disease between Sri Lankans in Oslo, Norway, and in Kandy, Sri Lanka. *Asia-Pac J Public Health* 2015;27:Np616–625.
[138]Andersen E, Ekelund U and Anderssen SA. Effects of reducing sedentary time on glucose metabolism in immigrant Pakistani men. *Med Sci Sports Exerc* 2015;47:775–781.
[139]Rabanal KS, Selmer RM, Igland J, et al. Ethnic inequalities in acute myocardial infarction and stroke rates in Norway 1994-2009: a nationwide cohort study (CVDNOR). *BMC Public Health* 2015;15:1073.
[140]Gele AA, Pettersen KS, Kumar B, et al. Diabetes risk by length of residence among Somali WOMEN in Oslo area. *J Diabetes Res*. 2016;2016:5423405.
[141]Rabanal KS, Meyer HE, Tell GS, et al. Can traditional risk factors explain the higher risk of cardiovascular disease in South Asians compared to Europeans in Norway and New Zealand? Two cohort studies. *BMJ Open* 2017;7:e016819.
[142]Ahmed SH, Meyer HE, Kjøllesdal MK, et al. Prevalence and predictors of overweight and obesity among Somalis in Norway and Somaliland: a comparative study. *J Obes* 2018;2018:4539171.
[143]Toftemo I, Jenum AK, Lagerløv P, et al. Contrasting patterns of overweight and thinness among preschool children of different ethnic groups in Norway, and relations with maternal and early life factors. *BMC Public Health* 2018;18:1056.
[144]Abuelmagd W, Håkonsen H, Mahmood KQ, et al. Living with diabetes: personal interviews with Pakistani women in Norway. *J Immigr Minority Health* 2018;20:848–853.
[145]Tran AT, Berg TJ, Gjelsvik B, et al. Ethnic and gender differences in the management of type 2 diabetes: a cross-sectional study from Norwegian general practice. *BMC Health Serv Res* 2019;19:904.
[146]Winje BA, Oftung F, Korsvold GE, et al. Screening for tuberculosis infection among newly arrived asylum seekers: Comparison of QuantiFERON®TB Gold with tuberculin skin test. *BMC Infect Dis* 2008;8:65.
[147]Harstad I, Heldal E, Steinshamn SL, et al. Tuberculosis screening and follow-up of asylum seekers in Norway: a cohort study. *BMC Public Health* 2009;9:141.
[148]Harstad I, Heldal E, Steinshamn SL, et al. Screening and treatment of latent tuberculosis in a cohort of asylum seekers in Norway. *Scand J Public Health* 2009;38:275–282.
[149]Krogh K, Surén P, Mengshoel AT. Tuberculosis among children in Oslo, Norway, from 1998 to 2009. *Scand J Infect Dis*. 2010;42:866–872.
[150]Harstad I, Jacobsen GW, Heldal E, et al. The role of entry screening in case finding of tuberculosis among asylum seekers in Norway. *BMC Public Health* 2010;10:670.
[151]Harstad I, Winje BA, Heldal E, et al. Predictive values of QuantiFERON-TB Gold testing in screening for tuberculosis disease in asylum seekers. *Int J Tuberc Lung Dis* 2010;14:1209–1211.
[152]Bjerke SEY, Holter E, Vangen S, et al. Sexually transmitted infections among Pakistani pregnant women and their husbands in Norway. *Int J Womens Health* 2010;2:303–309.
[153]Sagbakken M, Bjune GA and Frich JC. Experiences of being diagnosed with tuberculosis among immigrants in Norway – factors associated with diagnostic delay: a qualitative study. *Scand J Public Health* 2010;38:283–290.
[154]Hassan N, Siddiqui AR, Abbas Z, et al. Clinical profile and HLA typing of autoimmune hepatitis from Pakistan. *Hepat Mon* 2013;13:e13598-e.
[155]Pullar ND, Steinum H, Tonby K, et al. Low prevalence of positive interferon-gamma tests in HIV-positive long-term immigrants in Norway. *Int J Tuberc Lung Dis* 2014;18:180–187.
[156]Harstad I, Henriksen AH and Sagvik E. Collaboration between municipal and specialist public health care in tuberculosis screening in Norway. *BMC Health Serv Res* 2014;14:238.
[157]Guzman Herrador BR, Rønning K, Borgen K, et al. Description of the largest cluster of tuberculosis notified in Norway 1997–2011: is the Norwegian tuberculosis control programme serving its purpose for high risk groups? *BMC Public Health* 2015;15:367.
[158]Jensenius M, Winje BA, Blomberg B, et al. Multidrug-resistant tuberculosis in Norway: a nationwide study, 1995–2014. *Int J Tuberc Lung Dis* 2016;20:786–792.
[159]Haukaas FS, Arnesen TM, Winje BA, et al. Immigrant screening for latent tuberculosis in Norway: a cost-effectiveness analysis. *Eur J Health Econ* 2017;18:405–415.
[160]Di Ruscio F, Bjørnholt JV, Leegaard TM, et al. MRSA infections in Norway: a study of the temporal evolution, 2006–2015. *PloS One* 2017;12:e0179771–e.
[161]Asfeldt A, Lind Bratlien D, Brekken A, et al. When Europe's back door stood open. *Tidsskr Nor Legeforen* 2018;138.
[162]Danielsen AS, Elstrøm P, Arnesen TM, et al. Targeting TB or MRSA in Norwegian municipalities during 'the refugee crisis' of 2015: a framework for priority setting in screening. *Eurosurveillance* 2019;24:1800676.
[163]Festvåg LV, Stanghelle JK, Gilhus NE, et al. Polio and post-polio syndrome in non-Western immigrants: a new challenge for the healthcare system in Norway. *J Rehabil Med*. 2019;51:861–868.
[164]Winje BA, Grøneng GM, White RA, et al. Immigrant screening for latent tuberculosis infection: numbers needed to test and treat, a Norwegian population-based cohort study. *BMJ Open* 2019;9:e023412.
[165]Nordstoga I, Drage M, Steen TW, et al. Wanting to or having to – a qualitative study of experiences and attitudes towards migrant screening for tuberculosis in Norway. *BMC Public Health* 2019;19:796.
[166]Gele AA, Kumar B, Hjelde KH, et al. Attitudes toward female circumcision among Somali immigrants in Oslo: a qualitative study. *Int J Womens Health* 2012;4:7–17.
[167]Gele AA, Johansen EB and Sundby J. When female circumcision comes to the West: attitudes toward the practice among Somali Immigrants in Oslo. *BMC Public Health* 2012;12:697.
[168]Schultz J-H and Lien I-L. Meaning-making of female genital cutting: children's perception and acquired knowledge of the ritual. *Int J Womens Health* 2013;5:165–175.
[169]Gele AA, Sagbakken M and Kumar B. Is female circumcision evolving or dissolving in Norway? A qualitative study on attitudes toward the practice among young Somalis in the Oslo area. *Int J Womens Health* 2015;7:933–943.
[170]Ziyada MM, Norberg-Schulz M and Johansen REB. Estimating the magnitude of female genital mutilation/cutting in Norway: an extrapolation model. *BMC Public Health* 2016;16:110.
[171]Leinonen MK, Campbell S, Ursin G, et al. Barriers to cervical cancer screening faced by immigrants: a registry-based study of 1.4 million women in Norway. *Eur J Public Health* 2017;27:873–879.
[172]Møen KA, Kumar B, Qureshi S, et al. Differences in cervical cancer screening between immigrants and nonimmigrants in Norway: a primary healthcare register-based study. *Eur J Cancer Prev* 2017;26:521–527.
[173]Gele AA, Qureshi SA, Kour P, et al. Barriers and facilitators to cervical cancer screening among Pakistani and Somali immigrant women in Oslo: a qualitative study. *Int J Womens Health* 2017;9:487–496.
[174]Johansen REB. Undoing female genital cutting: perceptions and experiences of infibulation, defibulation and virginity among Somali and Sudanese migrants in Norway. *Cult Health Sex* 2017;19:528–542.

Table 1. (Continued)

[175]Johansen REB. Virility, pleasure and female genital mutilation/cutting. A qualitative study of perceptions and experiences of medicalized defibulation among Somali and Sudanese migrants in Norway. *Reprod Health* 2017;14:25.
[176]Mbanya VN, Gele AA, Diaz E, et al. Health care-seeking patterns for female genital mutilation/cutting among young Somalis in Norway. *BMC Public Health* 2018;18:517.
[177]Møen KA, Terragni L, Kumar B, et al. Cervical cancer screening among immigrant women in Norway – the healthcare providers' perspectives. *Scand J Prim Health Care*.2018;36:415–422.
[178]Bhargava S, Tsuruda K, Moen K, et al. Lower attendance rates in immigrant versus non-immigrant women in the Norwegian Breast Cancer Screening Programme. *J Med Screen* 2018;25:155–161.
[179]Johansen REB. Blurred transitions of female genital cutting in a Norwegian Somali community. *PloS One* 2019;14:e0220985.
[180]Bhargava S, Akslen LA, Bukholm IRK, et al. Performance measures among non-immigrants and immigrants attending BreastScreen Norway: a population-based screening programme. *Eur Radiol* 2019;29:4833–4842.
[181]Qureshi SA, Gele A, Kour P, et al. A community-based intervention to increase participation in cervical cancer screening among immigrants in Norway. *BMC Med Res Methodol* 2019;19:147.
[182]Meyer HE, Holvik K, Lofthus CM, et al. Vitamin D status in Sri Lankans living in Sri Lanka and Norway. *Br J Nutr* 2008;99:941–944.
[183]Madar AA, Klepp KI and Meyer HE. Effect of free vitamin D(2) drops on serum 25-hydroxyvitamin D in infants with immigrant origin: a cluster randomized controlled trial. *Eur J Clin Nutr* 2009;63:478–484.
[184]Madar AA, Stene LC and Meyer HE. Vitamin D status among immigrant mothers from Pakistan, Turkey and Somalia and their infants attending child health clinics in Norway. *Br J Nutr* 2009;101:1052–1058.
[185]Knutsen KV, Brekke M, Gjelstad S, et al. Vitamin D status in patients with musculoskeletal pain, fatigue and headache: a cross-sectional descriptive study in a multi-ethnic general practice in Norway. *Scand J Prim Health Care* 2010;28:166–171.
[186]Eggemoen ÅR, Knutsen KV, Dalen I, et al. Vitamin D status in recently arrived immigrants from Africa and Asia: a cross-sectional study from Norway of children, adolescents and adults. *BMJ Open* 2013;3:e003293.
[187]Knutsen KV, Madar AA, Lagerløv P, et al. Does vitamin D improve muscle strength in adults? A randomized, double-blind, placebo-controlled trial among ethnic minorities in Norway. *J Clin Endocrinol Metab* 2014;99:194–202.
[188]Wium C, Eggesbø HB, Ueland T, et al. Adipose tissue distribution in relation to insulin sensitivity and inflammation in Pakistani and Norwegian subjects with type 2 diabetes. *Scand J Clin Lab Invest* 2014;74:700–707.
[189]Rasmussen T, Yap SE, Stray-Pedersen B, et al. HLA associated type 1 diabetes risk in children of Pakistani migrants to Norway. *Med Hypotheses* 2014;83:664–667.
[190]Madar AA, Knutsen KV, Stene LC, et al. Effect of vitamin D3 supplementation on glycated hemoglobin (HbA1c), fructosamine, serum lipids, and body mass index: a randomized, double-blinded, placebo-controlled trial among healthy immigrants living in Norway. *BMJ Open Diabetes Res Care* 2014;2:e000026.
[191]Madar AA, Knutsen KV, Stene LC, et al. Effect of vitamin D(3)-supplementation on bone markers (serum P1NP and CTX): a randomized, double blinded, placebo controlled trial among healthy immigrants living in Norway. *Bone Rep* 2015;2:82–88.
[192]Dzidzonu DK, Skrivarhaug T, Joner G, et al. Ethnic differences in the incidence of type 1 diabetes in Norway: a register-based study using data from the period 2002-2009. *Pediatr Diabetes* 2016;17:337–341.
[193]Meyer HE, Skram K, Berge IA, et al. Nutritional rickets in Norway: a nationwide register-based cohort study. *BMJ Open* 2017;7:e015289.
[194]Madar AA, Gundersen TE, Haug AM, et al. Vitamin D supplementation and vitamin D status in children of immigrant background in Norway. *Public Health Nutr* 2017;20:2887–2892.
[195]Knutsen KV, Madar AA, Brekke M, et al. Effect of Vitamin D on thyroid autoimmunity: a randomized, double-blind, controlled trial among ethnic minorities. *J Endocr Soc* 2017;1:470–479.
[196]Madar AA, Meltzer HM, Heen E, et al. Iodine status among Somali immigrants in Norway. *Nutrients* 2018;10.
[197]Log T, Skurtveit S, Tverdal A, et al. Dispensing of prescribed analgesics in Norway among young people with foreign- or Norwegian-born parents. *Scand J Pain* 2011;2:36–44.
[198]Borchgrevink P. Trends in analgesic drug use evaluated by national prescription data bases: differences between immigrants and native citizens of Norway. *Scand J Pain* 2011;2:34–35.
[199]Sverre BL, Solbrække KN and Eilertsen G. Stories of pain and health by elderly Pakistani women in Norway. *Scand J Public Health* 2014;42:96–97.
[200]Knutsen KV, Madar AA, Brekke M, et al. Effect of vitamin D on musculoskeletal pain and headache: a randomized, double-blind, placebo-controlled trial among adult ethnic minorities in Norway. *Pain* 2014;155:2591–2598.
[201]Teodorescu DS, Heir T, Siqveland J, et al. Chronic pain in multi-traumatized outpatients with a refugee background resettled in Norway: a cross-sectional study. *BMC Psychol* 2015;3:7.
[202]Nortvedt L, Hansen HP, Kumar BN, et al. Caught in suffering bodies: a qualitative study of immigrant women on long-term sick leave in Norway. *J Clin Nurs* 2015;24:3266–3275.
[203]Nortvedt L, Lohne V, Kumar BN, et al. A lonely life – a qualitative study of immigrant women on long-term sick leave in Norway. *Int J Nurs Stud* 2016;54:54–64.
[204]Nyen S and Tveit B. Symptoms without disease: exploring experiences of non-Western immigrant women living with chronic pain. *Health Care Women Int* 2018;39:322–342.
[205]Hasha W, Fadnes LT, Igland J, et al. Two interventions to treat pain disorders and post-traumatic symptoms among Syrian refugees: protocol for a randomized controlled trial. *Trials* 2019;20:784.
[206]Skeie MS, Espelid I, Riordan PJ, et al. Caries increment in children aged 3–5 years in relation to parents' dental attitudes: Oslo, Norway 2002 to 2004. *Community Dent Oral Epidemiol* 2008;36:441–450.
[207]Skaret E, Espelid I, Skeie MS, et al. Parental beliefs and attitudes towards child caries prevention: assessing consistency and validity in a longitudinal design. *BMC Oral Health* 2008;8:1.
[208]Wigen TI and Wang NJ. Caries and background factors in Norwegian and immigrant 5-year-old children. *Community Dent Oral Epidemiol* 2010;38:19–28.
[209]Skeie MS, Klock KS, Haugejorden O, et al. Tracking of parents' attitudes to their children's oral health-related behavior-Oslo, Norway, 2002–04. *Acta Odontol Scand* 2010;68:49–56.
[210]Høyvik AC, Lie B, Grjibovski AM and Willumsen T. Oral health challenges in refugees from the Middle East and Africa: a comparative study. *J Immigr Minority Health* 2019;21:443–450.
[211]Amundsen EJ. Low level of alcohol drinking among two generations of non-Western immigrants in Oslo: a multi-ethnic comparison. *BMC Public Health* 2012;12:535.
[212]Vedøy TF. The role of education for current, former and never-smoking among non-western immigrants in Norway. Does the pattern fit the model of the cigarette epidemic? *Ethn Health* 2013;18:190–210.
[213]Abebe DS, Hafstad GS, Brunborg GS, et al. Binge drinking, cannabis and tobacco use among ethnic Norwegian and ethnic minority adolescents in Oslo, Norway. *J Immigr Minority Health* 2015;17:992–1001.
[214]Skogen JC, Bøe T, Sivertsen B, et al. Use of alcohol, tobacco and illicit drugs among ethnic Norwegian and ethnic minority adolescents in Hordaland county, Norway: the youth@hordaland-survey. *Ethn Health* 2018;23:43–56.
[215]Smestad C, Sandvik L, Holmoy T, et al. Marked differences in prevalence of multiple sclerosis between ethnic groups in Oslo, Norway. *J Neurol* 2008;255:49–55.
[216]Berg-Hansen P, Smestad C, Sandvik L, et al. Increased disease severity in non-Western immigrants with multiple sclerosis in Oslo, Norway. *Eur J Neurol* 2013;20:1546–1552.
[217]Berg-Hansen P, Moen SM, Sandvik L, et al. Prevalence of multiple sclerosis among immigrants in Norway. *Mult Scler J* 2014;21:695–702.
[218]Boldingh MI, Maniaol A, Brunborg C, et al. Prevalence and clinical aspects of immigrants with myasthenia gravis in northern Europe. *Muscle Nerve* 2017;55:819–827.
[219]Latif F, Helgeland J, Bukholm G, et al. Ethnicity differences in breast cancer stage at the time of diagnosis in Norway. *Scand J Surg* 2015;104:248–253.

Table 1. (Continued)

[220]Thøgersen H, Møller B, Robsahm TE, et al. Comparison of cancer stage distribution in the immigrant and host populations of Norway, 1990–2014. *Int J Cancer* 2017;141:52–61.
[221]Hjerkind KV, Qureshi SA, Møller B, et al. Ethnic differences in the incidence of cancer in Norway. *Int J Cancer* 2017;140:1770–1780.
[222]Thøgersen H, Møller B, Robsahm TE, et al. Differences in cancer survival between immigrants in Norway and the host population. *Int J Cancer* 2018;143:3097–3105.
[223]Chen SL, Dahl C, Meyer HE, et al. Estimation of Salt Intake Assessed by 24-Hour Urinary Sodium Excretion among Somali Adults in Oslo, Norway. *Nutrients*. 2018;10.
[224]Henjum S, Caswell BL and Terragni L. 'I feel like I'm eating rice 24 hours a day, 7 days a week': dietary diversity among asylum seekers living in Norway. *Nutrients* 2019;11.
[225]Henjum S, Morseth MS, Arnold CD, et al. 'I worry if I will have food tomorrow': a study on food insecurity among asylum seekers living in Norway. *BMC Public Health* 2019;19:592.
[226]Diaz E, Poblador-Pou B, Gimeno-Feliu L-A, et al. Multimorbidity and its patterns according to immigrant origin. A nationwide register-based study in Norway. *PloS One* 2015;10:e0145233.
[227]Diaz E, Kumar BN, Gimeno-Feliu LA, et al. Multimorbidity among registered immigrants in Norway: the role of reason for migration and length of stay. *Trop Med Int Health* 2015;20:1805–1814.
[228]Hjellset VT, Ihlebæk CM, Bjørge B, et al. Health-related quality of life, subjective health complaints, psychological distress and coping in Pakistani immigrant women with and without the metabolic syndrome: the InnvaDiab-DEPLAN study on Pakistani immigrant women living in Oslo, Norway. *J Immigr Minority Health* 2011;13:732–741.
[229]Myhrvold T and Småstuen MC. Undocumented migrants' life situations: an exploratory analysis of quality of life and living conditions in a sample of undocumented migrants living in Norway. *J Clin Nurs* 2019;28:2073–2087.
[230]Falch JA. Epidemiology of fractures of the distal forearm in Oslo, Norway. *Acta Orthop Scand* 1983;54:291–295.
[231]Amundsen VV, Wie OB, Myhrum M, et al. The impact of ethnicity on cochlear implantation in Norwegian children. *Int J Pediatr Otorhinolaryngol* 2017;93:30–36.
[232]Kvaal SI and Haugen M. Comparisons between skeletal and dental age assessment in unaccompanied asylum seeking children. *J Forensic Odontostomatol* 2017;35:109–116.
[233]Diaz E, Kumar BN and Engedal K. Immigrant patients with dementia and memory impairment in primary health care in Norway: a national registry study. *Dement Geriatr Cogn Disord* 2015;39:321–331.
[234]Syse A, Dzamarija MT, Kumar BN, et al. An observational study of immigrant mortality differences in Norway by reason for migration, length of stay and characteristics of sending countries. *BMC Public Health* 2018;18:508.
[235]Riise ØR, Laake I, Bergsaker MAR, et al. Monitoring of timely and delayed vaccinations: a nation-wide registry-based study of Norwegian children aged < 2 years. *BMC Pediatr* 2015;15:180.
[236]Strømme EM, Haj-Younes J, Hasha W, et al. Health status and use of medication and their association with migration related exposures among Syrian refugees in Lebanon and Norway: a cross-sectional study. *BMC Public Health* 2020;20:341.
[237]Høye S and Severinsson E. Intensive care nurses' encounters with multicultural families in Norway: an exploratory study. *Intensive Crit Care Nurs* 2008;24:338–348.
[238]Varvin S and Aasland OG. [Physicians' attitude towards treating refugee patients]. *Tidsskr Nor Laegeforen* 2009;129:1488–1490.
[239]Claussen B, Dalgard OS and Bruusgaard D. Disability pensioning: can ethnic divides be explained by occupation, income, mental distress, or health? *Scand J Public Health* 2009;37:395–400.
[240]Stige SH and Sveaass N. Living in exile when disaster strikes at home. *Torture* 2010;20:76–91.
[241]Tran AT, Diep LM, Cooper JG, et al. Quality of care for patients with type 2 diabetes in general practice according to patients' ethnic background: a cross-sectional study from Oslo, Norway. *BMC Health Serv Res* 2010;10:145.
[242]Høye S and Severinsson E. Multicultural family members' experiences with nurses and the intensive care context: a hermeneutic study. *Intensive Crit Care Nurs* 2010;26:24–32.
[243]Hanssen I and Alpers LM. Utilitarian and common-sense morality discussions in intercultural nursing practice. *Nurs Ethics* 2010;17:201–211.
[244]Madar AA, Klepp KI and Meyer HE. The effect of tailor-made information on vitamin D status of immigrant mothers in Norway: a cluster randomized controlled trial. *Matern Child Nutr* 2011;7:92–99.
[245]Kale E, Finset A, Eikeland HL, et al. Emotional cues and concerns in hospital encounters with non-Western immigrants as compared with Norwegians: an exploratory study. *Patient Educ Couns* 2011;84:325–331.
[246]Håkonsen H and Toverud EL. Special challenges for drug adherence following generic substitution in Pakistani immigrants living in Norway. *Eur J Clin Pharmacol* 2011;67:193–201.
[247]Småland Goth UG and Berg JE. Migrant participation in Norwegian health care. A qualitative study using key informants. *Eur J Gen Pract* 2011;17:28–33.
[248]Guribye E. 'No God and no Norway': collective resource loss among members of Tamil NGO's in Norway during and after the last phase of the civil war in Sri Lanka. *Int J Ment Health Syst* 2011;5:18.
[249]Guribye E, Sandal GM and Oppedal B. Communal proactive coping strategies among Tamil refugees in Norway: a case study in a naturalistic setting. *Int J Ment Health Syst* 2011;5:9.
[250]Håkonsen H and Toverud EL. Cultural influences on medicine use among first-generation Pakistani immigrants in Norway. *Eur J Clin Pharmacol* 2012;68:171–178.
[251]Claussen B, Smeby L and Bruusgaard D. Disability pension rates among immigrants in Norway. *J Immigr Minority Health* 2012;14:259–263.
[252]Alnaes AH. Lost in translation: cultural obstructions impede living kidney donation among minority ethnic patients. *Camb Q Healthc Ethics* 2012;21:505–516.
[253]Håkonsen H, Lees K and Toverud EL. Cultural barriers encountered by Norwegian community pharmacists in providing service to non-Western immigrant patients. *Int J Clin Pharm* 2014;36:1144–1151.
[254]Debesay J, Harsløf I, Rechel B, et al. Facing diversity under institutional constraints: challenging situations for community nurses when providing care to ethnic minority patients. *J Adv Nurs* 2014;70:2107–2116.
[255]Debesay J, Harsløf I, Rechel B, et al. Dispensing emotions: Norwegian community nurses' handling of diversity in a changing organizational context. *Soc Sci Med* 2014;119:74–80.
[256]Alpers LM and Hanssen I. Caring for ethnic minority patients: a mixed method study of nurses' self-assessment of cultural competency. *Nurse Educ Today* 2014;34:999–1004.
[257]Elstad JI, Øverbye E and Dahl E. Prospective register-based study of the impact of immigration on educational inequalities in mortality in Norway. *BMC Public Health* 2015;15:364.
[258]Holmberg Fagerlund B, Pettersen KS, et al. Counseling immigrant parents about food and feeding practices: public health nurses' experiences. *Public Health Nurs* 2016;33:343–350.
[259]Gimeno-Feliu LA, Calderón-Larrañaga A, Prados-Torres A, et al. Patterns of pharmaceutical use for immigrants to Spain and Norway: a comparative study of prescription databases in two European countries. *Int J Equity Health* 2016;15:32.
[260]Gele AA, Pettersen KS, Torheim LE, et al. Health literacy: the missing link in improving the health of Somali immigrant women in Oslo. *BMC Public Health* 2016;16:1134.
[261]Straiton ML, Ledesma HML and Donnelly TT. A qualitative study of Filipina immigrants' stress, distress and coping: the impact of their multiple, transnational roles as women. *BMC Women's Health* 2017;17:72.
[262]Straiton ML and Myhre S. Learning to navigate the healthcare system in a new country: a qualitative study. *Scand J Prim Health Care* 2017;35:352–359.
[263]Hjörleifsson S, Hammer E and Díaz E. General practitioners' strategies in consultations with immigrants in Norway-practice-based shared reflections among participants in focus groups. *Fam Pract* 2018;35:216–221.
[264]Nosrati E, Jenum AK, Tran AT, et al. Ethnicity and place: the geography of diabetes inequalities under a strong welfare state. *Eur J Public Health* 2018;28:30–34.
[265]Lillehagen M and Lyngstad TH. Immigrant mothers' preferences for children's sexes: a register-based study of fertility behaviour in Norway. *Popul Stud (Camb)* 2018;72:91–107.
[266]Alpers LM. Distrust and patients in intercultural healthcare: a qualitative interview study. *Nurs Ethics* 2018;25:313–323.

Table 1. (Continued)

[267]Abuelmagd W, Osman BB, Håkonsen H, et al. Experiences of Kurdish immigrants with the management of type 2 diabetes: a qualitative study from Norway. *Scand J Prim Health Care* 2019;37:345–352.
[268]Leirbakk MJ, Magnus JH, Torper J, et al. Look to Norway: Serving new families and infants in a multiethnic population. *Infant Ment Health J* 2019;40:659–672.
[269]Arora S, Straiton M, Rechel B, et al. Ethnic boundary-making in health care: experiences of older Pakistani immigrant women in Norway. *Soc Sci Med* 2019;239:112555.
[270]Ziyada MM, Lien I-L and Johansen REB. Sexual norms and the intention to use healthcare services related to female genital cutting: a qualitative study among Somali and Sudanese women in Norway. *PloS One* 2020;15:e0233440.
[271]Oppedal B, Keles S, Cheah C, et al. Culture competence and mental health across different immigrant and refugee groups. *BMC Public Health* 2020;20:292.
[272]Martiny SE, Froehlich L, Soltanpanah J, et al. Young immigrants in Norway: The role of national and ethnic identity in immigrants' integration. *Scand J Psychol* 2020;61:312–324.
[273]Lien E, Nafstad P and Rosvold EO. Non-western immigrants' satisfaction with the general practitioners' services in Oslo, Norway. *Int J Equity Health* 2008;7:7.
[274]Ayazi T and Bøgwald KP. [Immigrants' use of out-patient psychiatric services]. *Tidsskr Nor Laegeforen* 2008;128:162–165.
[275]Sandvik H, Hunskaar S and Diaz E. Immigrants' use of emergency primary health care in Norway: a registry-based observational study. *BMC Health Serv Res* 2012;12:308.
[276]Diaz E and Kumar BN. Differential utilization of primary health care services among older immigrants and Norwegians: a register-based comparative study in Norway. *BMC Health Serv Res* 2014;14:623.
[277]Diaz E, Gimeno-Feliu L-A, Calderón-Larrañaga A, et al. Frequent attenders in general practice and immigrant status in Norway: a nationwide cross-sectional study. *Scand J Prim Health Care* 2014;32:232–240.
[278]Goth US, Hammer HL and Claussen B. Utilization of Norway's emergency wards: the second 5 years after the introduction of the patient list system. *Int J Environ Res Public Health* 2014;11:3375–3386.
[279]Straiton M, Reneflot A and Diaz E. Immigrants' use of primary health care services for mental health problems. *BMC Health Serv Res* 2014;14:341.
[280]Diaz E, Calderón-Larrañaga A, Prado-Torres A, et al. How do immigrants use primary health care services? A register-based study in Norway. *Eur J Public Health* 2015;25:72–78.
[281]Ruud SE, Aga R, Natvig B, et al. Use of emergency care services by immigrants – a survey of walk-in patients who attended the Oslo Accident and Emergency Outpatient Clinic. *BMC Emerg Med* 2015;15:25.
[282]Gele AA, Torheim LE, Pettersen KS, et al. Beyond culture and language: access to diabetes preventive health services among Somali women in Norway. *J Diabetes Res* 2015;2015:549795.
[283]Fadnes LT, Møen KA and Diaz E. Primary healthcare usage and morbidity among immigrant children compared with non-immigrant children: a population-based study in Norway. *BMJ Open* 2016;6:e012101.
[284]Ruud SE, Hjortdahl P and Natvig B. Is it a matter of urgency? A survey of assessments by walk-in patients and doctors of the urgency level of their encounters at a general emergency outpatient clinic in Oslo, Norway. *BMC Emerg Med* 2016;16:22.
[285]Elstad JI. Register study of migrants' hospitalization in Norway: world region origin, reason for migration, and length of stay. *BMC Health Serv Res* 2016;16:306.
[286]Aarseth S, Kongshavn T, Maartmann-Moe K, et al. Paperless migrants and Norwegian general practitioners. *Tidsskr Nor Laegeforen* 2016;136:911–913.
[287]Czapka EA and Sagbakken M. 'Where to find those doctors?' A qualitative study on barriers and facilitators in access to and utilization of health care services by Polish migrants in Norway. *BMC Health Serv Res* 2016;16:460.
[288]Tatara N, Kjøllesdal MK, Mirkovic J, et al. eHealth use among first-generation immigrants from Pakistan in the Oslo area, Norway, with focus on diabetes: survey protocol. *JMIR Res Protoc* 2016;5:e79.
[289]Ruud SE, Hjortdahl P and Natvig B. Reasons for attending a general emergency outpatient clinic versus a regular general practitioner – a survey among immigrant and native walk-in patients in Oslo, Norway. *Scand J Prim Health Care* 2017;35:35–45.
[290]Fadnes LT and Diaz E. Primary healthcare usage and use of medications among immigrant children according to age of arrival to Norway: a population-based study. *BMJ Open* 2017;7:e014641.
[291]Diaz E, Mbanya VN, Gele AA, et al. Differences in primary health care use among sub-Saharan African immigrants in Norway: a register-based study. *BMC Health Serv Res* 2017;17:509.
[292]Tatara N, Hammer HL, Andreassen HK, et al. The association between commonly investigated user factors and various types of eHealth use for self-care of type 2 diabetes: case of first-generation immigrants from Pakistan in the Oslo area, Norway. *JMIR Public Health Surveill* 2017;3:e68.
[293]Straiton ML, Ledesma HML and Donnelly TT. 'It has not occurred to me to see a doctor for that kind of feeling': a qualitative study of Filipina immigrants' perceptions of help seeking for mental health problems. *BMC Women's Health* 2018;18:73.
[294]Abebe DS, Elstad JI and Lien L. Utilization of somatic specialist services among psychiatric immigrant patients: the Norwegian patient registry study. *BMC Health Serv Res* 2018;18:852.
[295]Finnvold JE. How social and geographical backgrounds affect hospital admission with a serious condition: a comparison of 11 immigrant groups with native-born Norwegians. *BMC Health Serv Res* 2018;18:843.
[296]Sagbakken M, Spilker RS and Nielsen TR. Dementia and immigrant groups: a qualitative study of challenges related to identifying, assessing, and diagnosing dementia. *BMC Health Serv Res* 2018;18:910.
[297]Straiton M, Corbett K, Hollander A-C and Hauge LJ. Outpatient mental healthcare service use among women with migrant background in Norway: a national register study. *BMC Health Serv Res* 2019;19:944.
[298]Schein YL, Winje BA, Myhre SL, et al. A qualitative study of health experiences of Ethiopian asylum seekers in Norway. *BMC Health Serv Res* 2019;19:958.
[299]Tschirhart N, Diaz E and Ottersen T. Accessing public healthcare in Oslo, Norway: the experiences of Thai immigrant masseuses. *BMC Health Serv Res* 2019;19:722.
[300]Czapka EA, Gerwing J and Sagbakken M. Invisible rights: barriers and facilitators to access and use of interpreter services in health care settings by Polish migrants in Norway. *Scand J Public Health* 2019;47:755–764.
[301]Mbanya VN, Terragni L, Gele AA, et al. Access to Norwegian healthcare system – challenges for sub-Saharan African immigrants. *Int J Equity Health* 2019;18:125.
[302]Arfa S, Solvang PK, Berg B, et al. Disabled and immigrant, a double minority challenge: a qualitative study about the experiences of immigrant parents of children with disabilities navigating health and rehabilitation services in Norway. *BMC Health Serv Res* 2020;20:134.
[303]Mbanya VN, Terragni L, Gele AA, et al. Barriers to access to the Norwegian healthcare system among sub-Saharan African immigrant women exposed to female genital cutting. *PloS one* 2020;15:e0229770.
